# A highly conserved segmental duplication in the subtelomeres of *Plasmodium falciparum *chromosomes varies in copy number

**DOI:** 10.1186/1475-2875-7-46

**Published:** 2008-03-07

**Authors:** Bobo W Mok, Ulf Ribacke, Ellen Sherwood, Mats Wahlgren

**Affiliations:** 1 Department of Microbiology, Tumor and Cell Biology (MTC), Karolinska Institutet, SE-171 77 Stockholm, Sweden; 2 Swedish Institute for Infectious Disease Control (SMI), SE-171 82 Stockholm, Sweden; 3 Department of Cell and Molecular Biology (CMB), Karolinska Institutet, SE-171 77 Stockholm, Sweden

## Abstract

**Background:**

Segmental duplications (SD) have been found in genomes of various organisms, often accumulated at the ends of chromosomes. It has been assumed that the sequence homology in-between the SDs allow for ectopic interactions that may contribute to the emergence of new genes or gene variants through recombinatorial events.

**Methods:**

*In silico *analysis of the 3D7 *Plasmodium falciparum *genome, conducted to investigate the subtelomeric compartments, led to the identification of subtelomeric SDs. Sequence variation and copy number polymorphisms of the SDs were studied by DNA sequencing, real-time quantitative PCR (qPCR) and fluorescent *in situ *hybridization (FISH). The levels of transcription and the developmental expression of copy number variant genes were investigated by qPCR.

**Results:**

A block of six genes of >10 kilobases in size, including *var*, *rif*, *pfmc-2tm *and three hypothetical genes (*n-, o- *and *q-gene*), was found duplicated in the subtelomeric regions of chromosomes 1, 2, 3, 6, 7, 10 and 11 (SD1). The number of SD1 per genome was found to vary from 4 to 8 copies in between different parasites. The intragenic regions of SD1 were found to be highly conserved across ten distinct fresh and long-term cultivated *P. falciparum*. Sequence variation was detected in a ≈ 23 amino-acid long hypervariable region of a surface-exposed loop of PFMC-2TM. A hypothetical gene within SD1, the *n-gene*, encoding a PEXEL/VTS-containing two-transmembrane protein was found expressed in ring stage parasites. The *n-gene *transcription levels were found to correlate to the number of *n-gene *copies. Fragments of SD1 harbouring two or three of the SD1-genes (*o-gene, pfmc-2tm, q-gene*) were also found in the 3D7 genome. In addition a related second SD, SD2, of ≈ 55% sequence identity to SD1 was found duplicated in a fresh clinical isolate but was only present in a single copy in 3D7 and in other *P. falciparum *lines or clones.

**Conclusion:**

*Plasmodium falciparum *carries multiple sequence conserved SDs in the otherwise highly variable subtelomeres of its chromosomes. The uniqueness of the SDs amongst plasmodium species, and the conserved nature of the genes within, is intriguing and suggests an important role of the SD to *P. falciparum*.

## Background

*Plasmodium falciparum*, the causative agent of severe human malaria, carries a haploid nuclear genome of approximately 23 Mb distributed onto 14 chromosomes [1]. The subtelomeric regions of the chromosomes are adjacent to telomeric repeats and appear to be species-specific, highly polymorphic and to consist of a patchworks of repetitive blocks [2]. Many families of variable genes are located in these regions. In addition to the well-studied *var *[3-5], *rif *[6,7] and *stevor *[8] genes, other multi-gene families such as *etramp/sep *[9,10], *surf *[11], *pfmc-2tm *[12], *phist *[13] and *fikk *[14,15] have recently been identified, most of which share the trait of sub-telomeric localization. These genes encode polypeptides many of which have been predicted to be involved in immune-evasion [16].

Syntenic comparisons of the *Plasmodium chabaudi, Plasmodium berghei*, and *Plasmodium yoelii *genomes with that of *P. falciparum *have revealed a striking conservation within the central cores of the chromosomes, whilst the subtelomeric regions display vast genetic variation [17,18]. For example, about 78% of the orphan genes of the *P. falciparum *genome with no detectable orthologs in the rodent parasites are located subtelomerically [18], indicating that most of the genetic variability is derived in this compartment. Therefore, genomic and genetic analyses of the subtelomeric content might provide information of the evolutionary forces driving speciation and factors contributing to biological variation among malaria parasites.

The plasticity and dynamic nature of the subtelomeres allow genes located in these regions to evolve more rapidly than their centromeric counterparts. Functional roles of telomeres and subtelomeres in antigenic variation include reversible gene silencing mediated by telomere-protein complexes [19] and engagement in ectopic exchange with other chromosomal ends [20,21]. The subtelomeres are polymorphic but they are also highly ordered within the end structures [22] where each subtelomere harbours five subtelomeric blocks (SB1 – 5) [17]. Recent studies have shown that the SB-3 (pRepHind or *rep20*) plays a role in mediating the formation and/or stabilization of telomere clusters [23,24]. Such physical tethering of chromosomes promotes recombination between virulence genes located at the end of heterologous chromosomes [24,25].

Genetic events such as transpositions, deletions, translocations and segmental duplications enable rapid adaptation to new environments. In genomes of primates and humans, a segmental duplication has been defined as a duplication of a DNA segment equal to or longer than 1 kb with a high level of sequence identity (> 90%) between copies transposed to new locations [26,27]. Due to the sequence identity between duplicated sequences and the resulting potential of genetic recombination, segmental duplications have contributed to the emergence of new genes or gene variants and thereby to the total genetic variation of genomes [26-28]. Well-described examples of such genetic changes is the generation of the repertoire of olfactory receptors in humans [29,30] and disease resistance genes in plants [31]. Further, it was recently shown that duplications and deletions in the human genome often are population specific [32].

Previous comparative genomic hybridization (CGH) approaches have demonstrated copy number polymorphisms located within internal regions of the *P. falciparum *chromosomes. The suitability of the same approach to detect duplications/deletions in the subtelomeres is limited due to the high degree of sequence variability genes in these compartments exhibit [33-35]. Nevertheless, a DNA segment on the right end of chromosome 1, spanning the genes PFA0685c, PFA0690w and PFA0695c, was previously suggested to be duplicated in a fresh clinical isolate [35]. Further analysis of this segment revealed covered genes being paralogous to genes within a >10 kb segmental duplication in the 3D7 subtelomeres (eight copies). In this study, the gene content, sequence polymorphism and copy number variation of the SDs have been investigated in distinct clones and lines of the parasite. In addition, transcription levels were monitored in parasites harbouring different numbers of SDs.

## Materials and methods

### Genome information

Sequence information, chromosomal locations and transcriptional directions of genes in the 3D7 genome were obtained from the Plasmodium Genome Resource [36]. Sequences from the Hb3 and Dd2 sequencing projects were retrieved from the Microbial Sequencing Center, Broad Institute [37]. Sequences from the Ghanaian isolate and the It/FCR3 strain were downloaded from The Plasmodium genome project, Welcome Trust Sanger Institute [38]. The coverage of Hb3, Dd2 and the Ghanaian isolate were 8.07×, 7.13× and 8× respectively, whereas the coverage of the It strain was estimated to be 3.84× by averaging the sizes of Hb3, Dd2 and Ghanaian isolate genomes and comparing the number of reads sequenced for the four strains.

Sequence reads were aligned to the *n*-, *o*-, *pfmc-2tm *and *q*-genes using BLASTN without low complexity filtering. The identity cutoff was set to 95% with a minimum accepted length of an overlap of ≥ 36 bp. The number of bps aligning to the genes was compared to the length of each gene, which yielded an estimated coverage for each gene in each of the parasite lines. This gene specific coverage was subsequently compared to the total coverage for the strains and a copy number estimate was calculated.

### Graphical presentation of the subtelomeres

**Figure 1:**
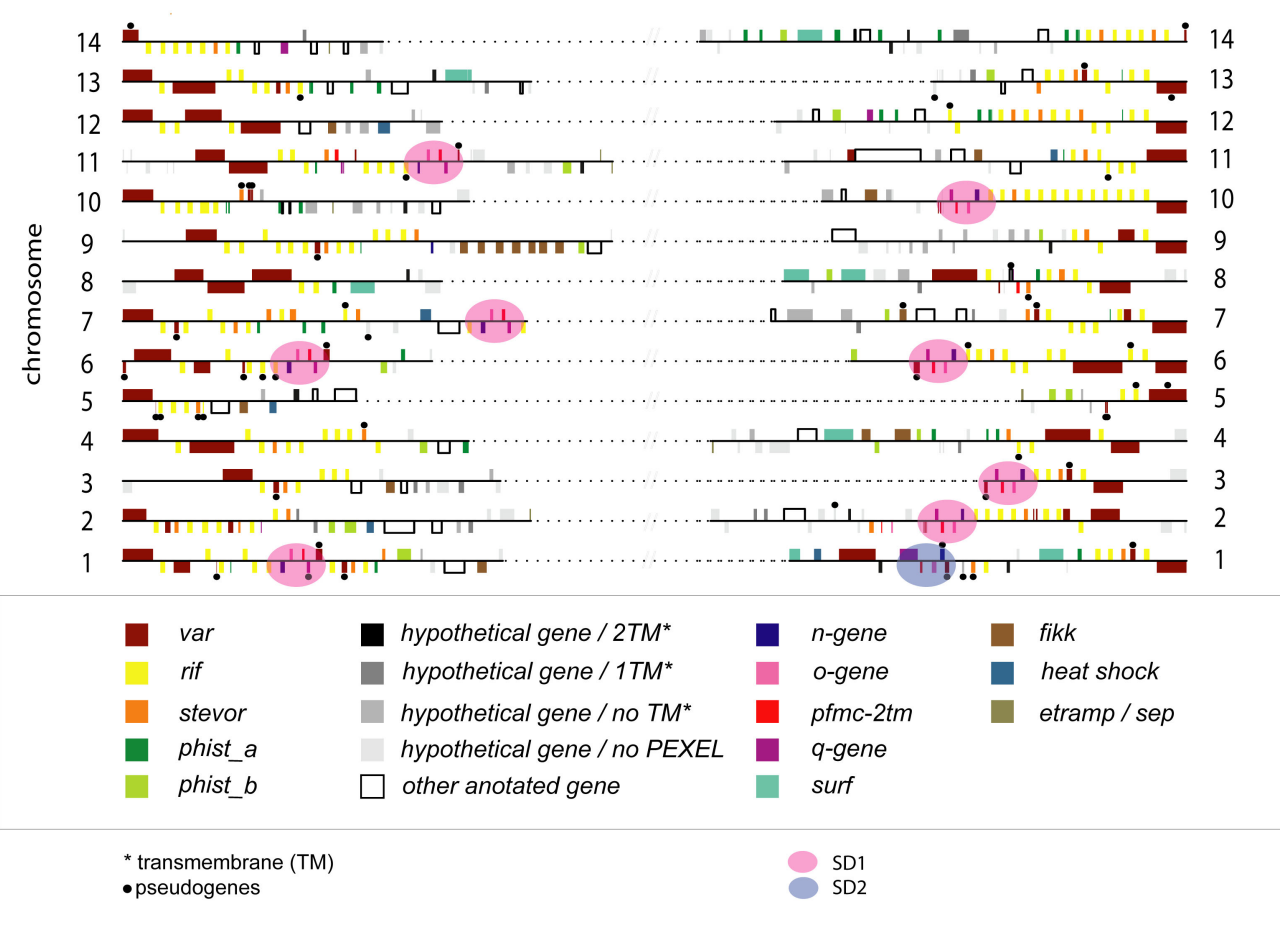
**High-resolution display of gene families in the subtelomeric compartment of *P. falciparum *3D7**. Subtelomeric genes are plotted according to their chromosomal positions and color labeled. For additional information see Additional File 1. The 8 segmental duplications SD1 are located in the subtelomeres of multiple chromosomes, and are here depicted in pink shaded ellipses. A second segmental duplication on chromosome 1 named SD2 is marked with a pale blue shaded ellipse.

A graphical output of all genes in the subtelomeric block 4–5 for all 14 chromosomes was generated (Figure 1). The boundaries of the subtelomeric ends were defined based on the whole genome synteny mapping of *P. falciparum *with rodent malaria parasites (*P. berghei*, *P. chabaudi *and *P. yoelii*) [18]. Subtelomeric gene-families are categorized into 18 groups (Additional File 1) and are displayed in different colors. Grouping of the subtelomeric genes was based on information from literature, the OrthoMCL Database [39] and/or protein features (possession of PEXEL/VTS domain and transmembrane regions) acquired from the Plasmodium database [36] where protein domains were predicted using HMM against the Pfam database, version 17.

### Nucleic acid extraction

Nucleic acids (gDNA and RNA) were extracted using either the Easy-DNA™ (Invitrogen) or the RNeasy^® ^(Qiagen) kits according to the recommendations of the suppliers. Total RNA was isolated from 3D7AH1, FCR3 and 7G8 at 8 to 28 hours post invasion with four-hour intervals for two consecutive parasite cycles. To ensure DNA-free RNA, the isolated RNA was treated with TURBO DNA-free™ DNAse (Ambion).

### PCR amplification and sequencing

Standard polymerase chain reaction (PCR) was used for the amplification of *n-*, *o-*, *pfmc-2tm*-, and *q-*genes of the SDs. Primers were designed based on the published 3D7 sequences: *n-gene*: forward 5'-TTT TTT TCA AGT AAG AGA TGC-3', reverse 5'-CCA CAA CCA CAC AAG AAG-3'; *o-gene*: forward 5'-CAA TAA ATA TAG CAA GTC G-3', reverse 5'-TAA ATC ATG TTC TGT GTG-3'; *pfmc-2tm*: forward 5'-ATC ATA CCA TAA TGG AGG-3', reverse 5'-ACC TAT TTT CAT GTC AGG-3' and *q-gene*: forward 5'-TGA AAA TAC CAA AGT ACC-3', reverse 5'-ATT GTA ATC CTT TAG CTC-3'. Amplification products were cloned into Topo vectors (TOPO TA cloning kit, Invitrogen) before transformation into TOP10 competent *E.coli*. DNA from at least four bacterial clones was sequenced for each target using M13 forward and reverse primers. ClustalW multiple alignments were performed thereafter, using BioEdit software version 7.0.5 (Tom hall, Ibis Therapeutics, Carlsbad, CA).

### Real-time quantitative PCR

Copy numbers relative to the 3D7AH1 parasite of the *n-gene*, PFA0675w, PFA0685c, PFA0690w and PFA0700c were determined for FCR3, 7G8, UAM25, HB3, Dd2, TM180 and TM284. Primers specific for the *n-gene *(5'-AGG GCA ATT GAT TTT AGC AGG TAT-3' and 5'-CAA AAC TAC TGA ATG CTA TAA ATG AAG GA-3'), PFA0675w (5'-TAT AAG ACC AAC TCT TTT CAT TTG TCT TTA C-3' and 5'-AAA ATC CTG TTG TAT GTA CGA TTA GCA T-3'), PFA0685c (5'-AAT ATA TAA CAA GTC GAG CAC TAA CGG A-3' and 5'-TCC TCT TAT TTG TGG ATT TTT ATT TCC-3'), PFA0690w (5'-ACC AAG AGC CTT GTG AAA CGA-3' and 5'-TTT CTT CCT TCT TCA GTT TTT TTG TG-3'), PFA0700c (5'-AGG AGA TTA CTA GCC GAA CCA CAC-3' and 5'-TTT ATG GGT TTT CAA TAT ATG TGA TTT GT-3') and the endogenous control gene PF10_0084 (5'-ACA ACG AAG CAA CAG GAG GTA GAT-3' and 5'-AGT CCA TCA ATA TAG CTC TTG GAA CAT A-3') were all designed using Primer Express 2.0 (Applied Biosystems) towards perfectly conserved stretches of the genes. Approximately 1 ng of DNA was used as template in quadruplicate amplification reactions in MicroAmp 96 well plates in 20 μl containing SYBR Green master mix and 300 nm of each primer. Amplifications were carried out in an ABI sequence detector 7500 (Applied Biosystems) for 40 cycles (95°C for 15 seconds and 60°C for 1 min). PCR-efficiencies of all primer-pairs were evaluated on dilution series of 3D7AH1 genomic DNA and found to be sufficiently close to obviate the need for any correction factor. Results were analysed using the ΔΔCt method (User bulletin 2, Applied Biosystems) based on the tested assumption that the target genes are amplified with the same efficiency as the endogenous control.

Total RNA was reversibly transcribed with SuperScript III Rnase H reverse transcriptase (Invitrogen), random hexamers and oligo(dT)_12–18 _(300 ng/μl and 25 ng/μl respectively, both from Invitrogen) for two hours at 50°C. For each cDNA synthesis reaction, a control reaction without reverse transcriptase was performed with identical amounts of template. For qPCR-based determination of *n-gene *transcription the same primers were used as listed above except for the endogenous control, where *seryl-tRNA synthetase *was employed. The primers were: 5'-TAT CAT CTC AAC AGG TAT CTA CAT CTC CTA-3' and 5'-TTT GAG AGT TAC ATG TGG TAT CAT CTT TT-3'. The amplification reactions were conducted as described above, with the only difference that 2 ng of template was used. Transcription levels were achieved by dividing the ${\bar{x}}_{Ctn-gene}$ with the ${\bar{x}}_{Ctseryl-tRNAsynthetase}$ for each strain and time point. The standard deviation of the quotient was calculated according to the User Bulletin 2, Applied Biosystems. Results were visualized as log_2 _transformed values and plotted using SigmaPlot 9.0 (Systat Software Inc.).

### Fluorescent in-situ hybridization

FISH targeting the *n-gene *was conducted according to previously described methodology [35]. The fluorescein labeled (Fluorescein-High Prime, Roche Applied Science) *n-gene *probe was generated from 3D7AH1 gDNA using the primers 5'-TTT TTT TCA AGT AAG AGA TGC-3' and 5'-CCA CAA CCA CAC AAG AAG-3'.

## Results

### Segmental duplications in the subtelomeres

**Figure 2:**
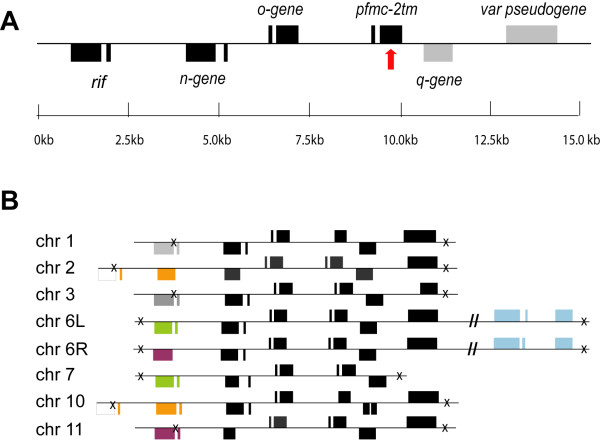
**Gene content and organization of the segmental duplicon SD1**. (A) Example of a typical SD1 containing six complete genes. Genes encoding PEXEL-containing proteins are depicted in black. A red arrow indicates the position of the hypervariable loop in *pfmc2tm*. (B) The SD1 exists in eight copies in the 3D7 genome with a slight variation in respect to the *rif *gene. Homologous *rif *copies, with > 95% sequence homology, in between the SD1s are shown with the same color. Crosses indicate breakpoints of each SD1.

Comparative analysis of the *P. falciparum *genome with rodent plasmodium species has disclosed synteny breaks at the boundaries of the subtelomeric compartments [18]. Here, we have analysed the subtelomeric gene content of the 3D7 genome by grouping the genes into families as shown in Figure 1. Eight homologous regions were found, all sharing the same genomic organization being located on seven chromosomes (Chromosomes 1, 2, 3, 6, 7, 10 and 11). This duplicated DNA segment (named SD1) was found to contain six genes: *rif, pfmc-2tm*, a *var *pseudogene and three hypothetical genes (*n-, o- and q-gene*) (Figure 2A). The breakpoints of these segmental duplicons vary slightly, with the 5' break point being either within or downstream with respect to the *rif *gene and the 3' break point being either upstream or downstream of the *var *pseudogene. The most extended duplicated loci (approximately 32 kb in size) are both located on chromosome 6, but on opposite chromosomal ends. Although the *rif *genes are not identical in-between the SD1, homologous *rif *copies can be found within all SD1 (Figure 2B). Most of the genes within SD1 encode PEXEL-containing export proteins, with the exception of the *q-gene *and the *var *pseudogenes (Additional File 2). SD1-fragments harbouring only two or three of the SD1- genes (*o-gene, pfmc-2tm, q-gene*) were also found in the 3D7 genome (Additional File 1).

**Figure 3:**
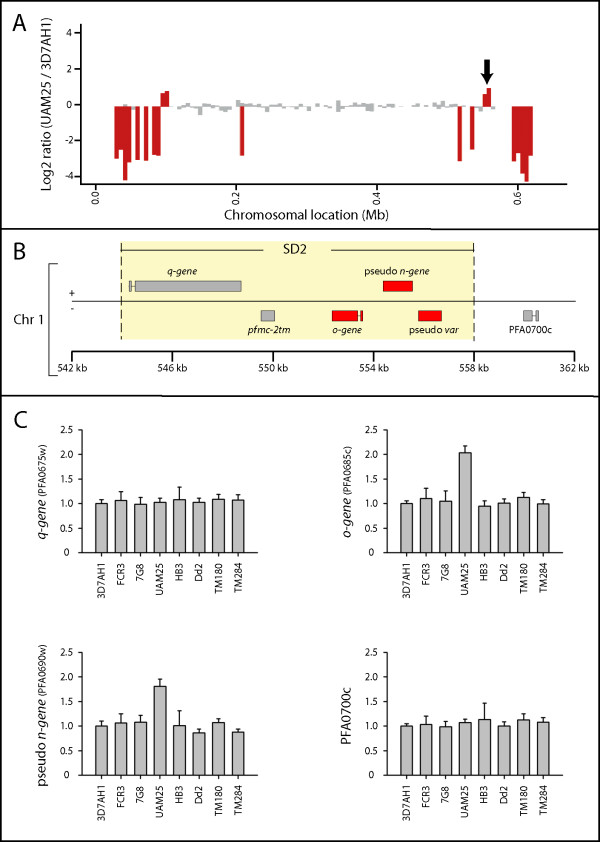
**Copy numbers of the SD2 in different *P. falciparum *strains**. (A) Ratio based differences (UAM25 over 3D7AH1) of microarray oligonucleotides mapped according to the gene locations on chromosome 1 in 3D7. A black arrow indicates the SD2 found duplicated in UAM25. (B) Genetic organization of the SD2 on the right arm of chromosome 1 in 3D7. Genes found duplicated in UAM25 relative to 3D7, according to CGH data and qPCR, are shown in red. (C) Copy numbers of the SD2 genes in different strains relative to 3D7 parasite confirmed by qPCR.

A previous CGH project from this laboratory revealed a subtelomeric gene segment (PFA0685c, PFA0690w and PFA0695c), located on the right end of chromosome 1 in the 3D7 strain, to be duplicated in a fresh clinical isolate (UAM25) [35] (Figure 3A). Further analysis indicates that this locus shares three of the same paralogous genes as SD1s described above, with the same gene order and orientation but with less sequence homology (55% identity). This SD was named SD2. Compared to the eight SD1, SD2 was found to carry the *n-gene *as a pseudogene and the *q-gene *(PFA0675w) was found to harbour RESA-like repeats and a DNAJ domain (PFAM database: PF0026; amino acid 1097–1160), which the *q-gene *of SD1 does not possess. PSI-BLAST analyses of the genes in the SD2 (converged at iteration 3) showed that the *q-gene *has orthologous genes in *P. vivax *and in rodent malaria parasites (*P. yoelii, P. chabaudi and P. berghei*). However, no orthologous genes could be identified for the other SD2 gene-members.

### Sequence variation within the segmental duplicons

To elucidate whether the sequence conservation of the SD1 remains across different *P. falciparum *parasites, we sequenced the *n-, o-, pfmc-2tm and q-gene *of five parasites originating from different geographical areas: FCR3 (The Gambia), TM180 (Thailand), 7G8 (Brazil), UAS31 and UAS39 (both from Uganda). In addition, sequence information for HB3 (Honduras) and Dd2 (Indochina) [37] and It (Brazil) [38] was retrieved for the analysis. ClustalW multiple alignments revealed that genes within the SD1s are of a high sequence identity (99%), with the exception of a ≈ 23 amino acid hypervariable loop within *pfmc-2tm *which is predicted to be surface-exposed [12,40]. Polymorphisms other than those of *pfmc-2tm *in the eight SD1s of 3D7 were mainly situated within repetitive sequence stretches of the intra- and intergenic regions. Comparisons of sequences to single nucleotide polymorphism (SNPs) data published recently [41] (Additional File 3) revealed four novel non-synonymous SNPs in the *n-gene*, and four non-synonymous and two synonymous SNPs in the *q-gene*.

### Copy number polymorphism of the segmental duplicons

**Figure 4:**
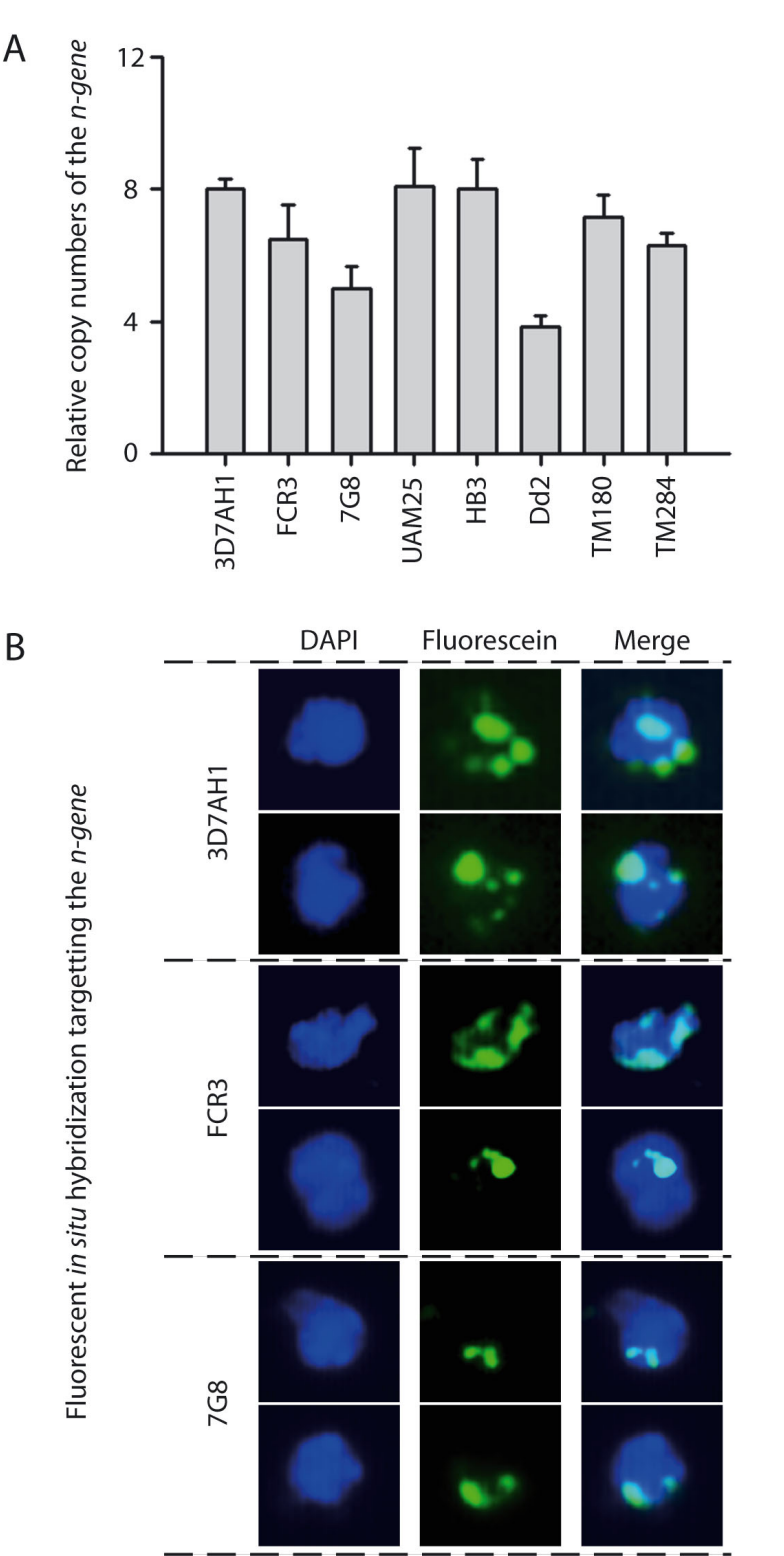
**Copy number polymorphisms of the *n-gene *in different *P. falciparum *strains and isolates**. (A) Copy numbers of the *n-gene *in different parasite lines relative to 3D7 detected by qPCR. (B) Visualization of copy numbers and localization of *n-gene *(green) in 3D7, FCR3 and 7G8. Distribution of fluorescent signals at the rim of the parasite nuclei (blue) confirms the position of the SD at the chromosomal ends.

Using the *n-gene *as a representative member of SD1, the SD1 copy number in different *P. falciparum *strains relative to the 3D7 parasite was estimated using qPCR. The genomes of HB3 and the clinical isolate (UAM25) were found to contain the same number of SD1 copies as 3D7 (n = 8), whereas Dd2 was found to carry ≤ 4 (Figure 4A). Comparable numbers of *pfmc-2tm *was previously reported for HB3 relative to 3D7 [40], signifying a copy number association between the *n-gene *and *pfmc-2tm*.

The results were further confirmed by fluorescent *in situ *hybridizations (FISH). In addition to a clear pattern of variable copy numbers (Figure 4A) most of the signals were distributed at the rim of the parasite-nuclei where chromosomal ends are known to tether [23], confirming the subtelomeric localization of the SD1s (Figure 4B).

The amplification of SD2 was also verified by qPCR targeting the pseudo *n-gene *(PFA0690w), as well as the adjacent genes, PFA0675w (pseudo *q-gene)*, PFA0685c (pseudo *o-gene) *and PFA0700c (Figure 3B). In contrast to SD1, the SD2 in UAM25 did not include PFA0675w (paralogous to the *q-gene*).

### Transcriptional analysis

The intraerythrocytic developmental expression of the genes in the SDs was previously studied using microarrays ([42] : E-MEXP-128) [43,44]. Only the *n-gene *was found significantly transcribed, with maximum expression in the ring stages. In addition, the pseudo *n-gene *(PFA0690w) of SD2 was found to be expressed, despite of its supposedly truncated ORF, with maximum transcript abundance at 36h post-invasion [44].

**Figure 5:**
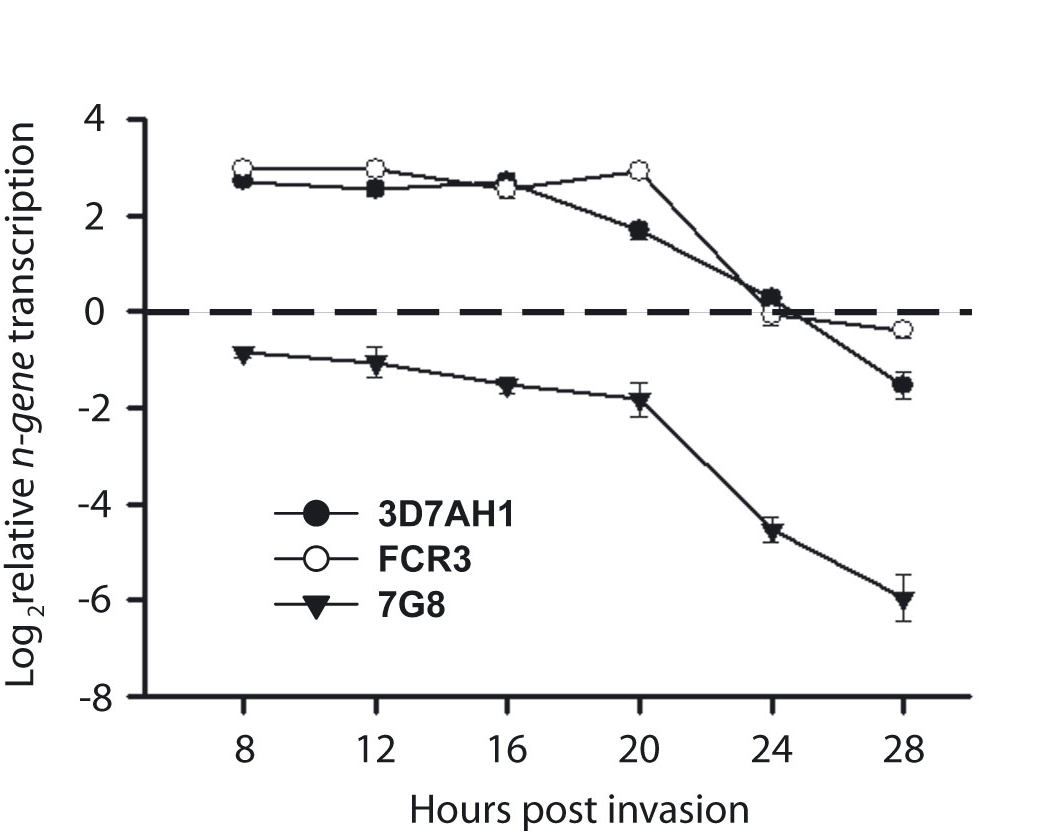
**Temporal and relative transcript abundance of the *n-gene *in 3D7, FCR3 and 7G8**. The transcript levels of the *n-gene*, in relation to the endogenous control gene *seryl-tRNA synthetase*, were measured during 8–28 hours post invasion. Data was log_2 _transformed and plotted at four-hour intervals for each particular parasite.

In order to investigate the impact of gene dosage on transcription levels, *n-gene *transcription was investigated for three parasites with varying numbers of SD1s. 3D7AH1, FCR3 and 7G8 parasites were harvested at 4-hour intervals from eight to 28 hours post-invasion and relative mRNA levels were studied by qPCR. The maximum level of transcription of the *n-gene *was found in ring-stage parasites, which coincides with previous transcription data [43,44]. A clear transcriptional difference was observed when comparing 3D7AH1 and 7G8, which carry eight and five copies in the genome, respectively, but similar level of transcription was found for 3D7AH1 and FCR3, although the latter carries fewer copies of the *n-gene *(Figure 5).

## Discussion

Genomic variability of *P. falciparum *has been shown to be of importance to the parasite and to underly the ability of local adaptation, antigenic variation and drug resistance. A substantial degree of genetic variation in between different *P. falciparum *strains is confined to the subtelomeric compartment [33,34], where families of variable genes and a number of repetitive regions are located. This paper reports the presence of a >10 kb segment duplicated and translocated onto multiple chromosomal ends. These paralogous regions span at least six genes and include members from the previously identified multigene families *rif*, *pfmc-2tm *and *var *as well as three families of hypothetical genes (*n-, o-, q-genes*) present as multiple homologous copies.

*Pfmc-2tm*, together with *rif *and *stevor*, belong to a large topological super family based on the presence of the erythrocyte-trafficking motif PEXEL/VTS [45-47] and two predicted transmembrane regions. While *rif *and *stevor *encode >150 RIFIN-related and >35 STEVOR-related proteins respectively, *pfmc-2tm *possesses a lower number of paralogous members (13 in the 3D7 genome) [12,40]. Sequences of *pfmc-2tm *genes are relatively conserved with differences located in a ≈ 23 amino acid region situated between the two predicted transmembrane regions. This hypervariable stretch is presumably forming a loop exposed on the infected red cell surface. Among the 13 *pfmc-2tm *genes in the 3D7 genome, 9 are located within, and the remaining adjacent to the SD. A possible explanation for this observation is that the *pfmc-2tm *gene family is expanded through segmental duplication. Subsequent modification of the duplicated genes, most likely by single nucleotide mutations within the variable loop region, may provide the diversity of this gene family. In contrast to *pfmc-2tm*, *var *gene diversity is probably generated mainly through ectopic recombination. This is facilitated by the neighbouring *rep20 *sequences, which mediate clustering of the telomeres and hence bring subtelomeric *var *genes into close proximity [21,24,25]. Although this study also suggests that members of the *rif *family might be expanded through segmental duplicative events, this still only represents a small fraction of this large gene family of more than 150 members [48].

Evolution of multigene families involves different rates of gene duplication, maintenance and loss, often accompanied by formation of pseudogenes [49]. Pseudogenes provide a record of how genomic DNA has been changed without such evolutionary pressure and can be used as a model for determining the underlying rates of nucleotide substitutions, insertions and deletions in the genome. The subtelomeric regions, due to their dynamic nature, are breeding grounds for generation of pseudogenes. In the 3D7 genome, 57 out of 73 pseudogenes belong to the three big multigene families (*rif, stevor *and *var*), of which the majority (44/57) are subtelomerically located (Figure 1, shown as). Except for SD1 on chromosome 7, all other SDs end with a *var *pseudogene. The sequence identity of these *var *pseudogenes is high, not only within the 3D7 but also across other genomes of *P. falciparum *parasites.

In addition to FISH and qPCR we also adopted an *in silico *strategy similar to the one used by Bailey *et al*. for locating copy number variations in the human genome [50] (see Material and Methods). Sequences from four strains (Hb3, Dd2, It and Ghanaian isolate) were downloaded and aligned to the *n-, o-, pfmc-2tm and q-gene *of the SDs. Different copy numbers of *n-, o-, pfmc-2tm *and *q-gene *were identified in all parasites (Additional File 4), but the copy numbers estimated with this method were lower compared to those obtained by qPCR. The most likely reason for this discrepancy is an incomplete assembly of these recently sequenced parasite genomes.

The *n-gene *belongs to the same PEXEL-containing two-transmembrane superfamily as *rif*, *stevor *and *pfmc-2tm *(Additional File 2). Previous microarray data from isogenic clones of 3D7 revealed that the *n-gene *is the only gene in the SD1 that is transcribed during the intraerythrocytic cycle. The transcription levels of the *n-gene *correlated in part with the copy number abundance (in 3D7 vs. 7G8 but not in 3D7 vs. FCR3). The gene-copy number may be one of the reasons for a relatively low level of *n-gene *transcription in Dd2 and high levels in HB3 as shown by Llinas *et al *[51]. Taken together it seems that the abundance of the *n-gene *affects the levels of mRNA.

The presence of four to eight copies of SD1 in all parasites studied and up to two copies of a second segmental duplication (SD2) in a freshly isolated parasite suggests that segmental duplications do occur in *P. falciparum *and that they are of biological importance *in vivo*. It may be that the SDs are part of a transposon-like system in *P. falciparum *but this remains to be investigated.

## Conclusion

*Plasmodium falciparum *carries multiple SD in the subtelomeres of its chromosomes. The unique presence of the SDs in *P. falciparum *compared to other Plasmodium species and the conserved nature of the genes within, suggests a functional role of the SDs to *P. falciparum*.

## Abbreviations

FISH, fluorescent *in situ *hybridization; PEXEL/VTS, Plasmodium export element or the vacuolar transport signal; qPCR, real-time quantitative PCR; SD, segmental duplication.

## Authors' contributions

BWM carried out the design of the study, sequencing, data analysis and wrote the manuscript. UR designed and carried out the qPCR and FISH and helped to finalize the manuscript. ES was involved in the *in silico *work on copy number predictions. MW participated in the study design and helped to finalize the manuscript. All authors have read and approved the final manuscript.

## Supplementary Material

Additional file 1 Groupings of the subtelomeric genes in 3D7 genome. Click here for file

Additional file 2 Topology of proteins encoded by the genes in the SDs. Red bars show transmembrane regions predicted by TMHMM; PEXEL motifs are indicated by green bars. Click here for file

Additional file 3 SNPs in *n-, o-, pfmc-2tm *and *q-gene *found in different *P.f*. strains. Click here for file

Additional file 4 Copy number estimation of SD genes using BLASTN. Click here for file
