# Supplementary material for: A highly conserved segmental duplication in the subtelomeres of Plasmodium falciparum chromosomes varies in copy number
Source: Malar J. 2008 Mar 7;7:46. doi: 10.1186/1475-2875-7-46 (PMC2279139; doi:10.1186/1475-2875-7-46)
Supplement: Additional file 1 — Groupings of the subtelomeric genes in 3D7 genome. [file 1475-2875-7-46-S1.pdf]

Additional File 1. Groupings of the subtelomeric genes in 3D7 genome

Rank	PlasmoDB ID	location	location	size	upstream	annotation	Group	Pseudogene
1	PFA0005w	29733	37349	7616	1856	erythrocyte membrane protein 1 (PfEMP1)	1	
2	PFA0010c	39205	40430	1225	2160	rifin	2	
3	PFA0015c	42590	46730	4140	3856	var-like protein	1	
4	PFA0020w	50586	51859	1273	1533	rifin	2	
5	PFA0025c	53392	53503	111	498	VAR fragment, pseudogene	1	1
6	PFA0030c	54001	55229	1228	1684	rifin	2	
7	PFA0035c	56913	57116	203	2889	hypothetical protein (truncated)	4	
8	PFA0040w	60005	61236	1231	1184	rifin	2	
9	PFA0045c	62420	63633	1213	2417	rifin	2	
10	PFA0050c	66050	67222	1172	2315	rifin	2	
11	PFA0055c	69537	70650	1113	1207	hypothetical protein, conserved in P. falciparum	n-gene	
12	PFA0060w	71857	72659	802	2362	hypothetical protein, conserved in P. falciparum	o-gene	
13	PFA0065w	75021	75599	578	616	hypothetical protein, conserved in P. falciparum	pfmc-2tm	
14	PFA0070c	76215	77042	827	1432	pseudogene, P. falciparum-specific conserved gene family	q-gene	1
15	PFA0075w	78474	80125	1651	1873	VAR-fragment, pseudogene	1	1
16	PFA0080c	81998	83339	1341	1685	rifin	2	
17	PFA0085c	85024	86385	1361	1051	truncated var-related protein	1	1
18	PFA0090c	87436	88410	974	2065	stevor	3	
19	PFA0095c	90475	91653	1178	1693	rifin	2	
20	PFA0100c	93346	94066	720	1191	hypothetical protein (truncated)	4	
21	PFA0105w	95257	96046	789	3006	stevor	3	
22	PFA0110w	99052	102515	3463	2421	ring-infected erythrocyte surface antigen precursor	5	
23	PFA0115w	104936	105441	505	1988	hypothetical protein	8	
24	PFA0120c	107429	108580	1151	2404	hypothetical protein	9	
25	PFA0125c	110984	116033	5049	3242	Ebl-1 like protein, putative	13	
26	PFA0130c	119275	121648	2373	3104	FIKK1	11	
27	PFA0135w	124752	125719	967		hypothetical protein	9	
28	PFA0655w	516313	519004	2691	3525	SURFIN, surface-associated interspersed gene	10	
29	PFA0660w	522529	524301	1772	4528	dnj1/sis1 family	12	
30	PFA0665w	528829	538073	9244	758	hypothetical protein	1	
31	PFA0670c	538831	539747	916	4455	hypothetical protein (=MAL13P1.61)	6	
32	PFA0675w	544202	548705	4503	614	hypothetical protein (C-terminus RESA-like with DNAJ domain)	q-gene	
33	PFA0680c	549319	549879	560	2419	hypothetical protein, conserved in P. falciparum	pfmc-2tm	
34	PFA0685c	552298	553435	1137	927	hypothetical protein, conserved in P. falciparum	o-gene	
35	PFA0690w	554362	555519	1157	83	pseudogene, P falciparum specific gene family	n-gene	1
36	PFA0695c	555602	556651	1049	3272	VAR like pseudogene	1	1
37	PFA0700c	559923	560430	507	1766	hypothetical protein, conserved in P. falciparum, pseudogene	8	1
38	PFA0705c	562196	563256	1060	2169	STEVOR pseudogene	3	1
39	PFA0710c	565425	566564	1139	4642	rifin	2	
40	PFA0715c	571206	571993	787	1715	hypothetical protein (= MAL8P1.160, MAL8P1.161)	6	
41	PFA0720w	573708	574404	696	5020	hypothetical protein	9	
42	PFA0725w	579424	585509	6085	810	SURFIN, surface-associated interspersed gene	10	
43	PFA0730c	586319	586588	269	2523	hypothetical protein	9	

Rank	PlasmoDB ID	location	location	size	upstream	annotation	Group	Pseudogene
44	PFA0735w	589111	590114	1003	3455	hypothetical protein	4	
45	PFA0740w	593569	594907	1338	1915	rifin	2	
46	PFA0745w	596822	597993	1171	2192	rifin	2	
47	PFA0750w	600185	601192	1007	1217	stevor	3	
48	PFA0755w	602409	603712	1303	2170	VAR related pseudogene	1	1
49	PFA0760w	605882	607251	1369	1859	rifin	2	
50	PFA0765c	609110	616613	7503		erythrocyte membrane protein 1 (PfEMP1)	1	
51	PFB0010w	25232	31168	5936	1862	erythrocyte membrane protein 1 (PfEMP1)	1	
52	PFB0015c	33030	34259	1229	1668	rifin	2	
53	PFB0020c	35927	37249	1322	1038	erythrocyte membrane protein 1 (PfEMP1), truncated	1	
54	PFB0025c	38287	39303	1016	2212	stevor, putative	3	
55	PFB0030c	41515	42858	1343	2428	rifin	2	
56	PFB0035c	45286	46800	1514	2123	rifin	2	
57	PFB0040c	48923	50147	1224	1695	rifin	2	
58	PFB0045c	51842	53124	1282	1294	erythrocyte membrane protein 1 (PfEMP1), truncated	1	
59	PFB0050c	54418	54936	518	2408	stevor isoform gam beta	3	
60	PFB0055c	57344	58421	1077	1686	rifin	2	
61	PFB0056c	60107	60319	212	2838	hypothetical protein, truncated	q-gene	
62	PFB0060w	63157	64376	1219	2174	rifin	2	
63	PFB0065w	66550	67545	995	1543	stevor, putative	3	
64	PFB0070w	69088	69771	683	3670	hypothetical protein (=PFA0700c)	7	
65	PFB0075c	73441	74396	955	2855	hypothetical protein	7	
66	PFB0080c	77251	78808	1557	2483	hypothetical protein	5	
67	PFB0085c	81291	84165	2874	2667	hypothetical protein	5	
68	PFB0090c	86832	88633	1801	2685	hypothetical protein, conserved	12	
69	PFB0095c	91318	98838	7520	4547	erythrocyte membrane protein 3	13	
70	PFB0100c	103385	105796	2411	3768	knob associated histidine-rich protein	13	
71	PFB0105c	109564	110580	1016	1967	hypothetical protein	8	
72	PFB0106c	112547	113682	1135	3543	hypothetical protein	7	
73	PFB0110w	117225	118235	1010	2289	hypothetical protein	9	
74	PFB0115w	120524	124102	3578	3892	hypothetical protein	9	
75	PFB0120w	127994	128314	320		etramp / sep	14	
76	PFB0925w	820427	822539	2112	1284	hypothetical protein	9	
77	PFB0926c	823823	824862	1039	6325	hypothetical protein	9	
78	PFB0930w	831187	832003	816	1800	hypothetical protein	7	
79	PFB0932w	833803	834686	883	4155	hypothetical protein	7	
80	PFB0935w	838841	844114	5273	746	cytoadherence linked asexual protein 2	13	
81	PFB0946c	844860	845837	977	1650	hypothetical protein	9	
82	PFB0950w	847487	849117	1630	2305	hypothetical protein	9	
83	PFB0951w	851422	851960	538	3083	hypothetical protein	9	1
84	PFB0953w	855043	855736	693	1697	hypothetical protein	6	
85	PFB0954c	857433	858043	610	412	hypothetical protein	9	
86	PFB1070w	858455	859069	614	1284	hypothetical protein	9	
87	PFB0955w	860353	861502	1149	1882	stevor, degenerate, putative	3	
88	PFB0960c	863384	863626	242	2288	hypothetical protein	pfmc-2tm	
89	PFB0965c	865914	866324	410	3182	hypothetical protein	o-gene	

Rank	PlasmoDB ID	location	location	size	upstream	annotation	Group	Pseudogene
90	PFB0970c	869506	871122	1616	2007	hypothetical protein (= PFB0926C)	9	
91	PFB0972w	873129	873317	188	598	hypothetical protein	9	
92	PFB0973c	873915	874109	194	20	hypothetical protein	9	
93	PFB0974c	874129	874323	194	220	erythrocyte membrane protein 1 (PfEMP1), truncated, degenerate	1	
94	PFB0975c	874543	874809	266	154	erythrocyte membrane protein 1 (PfEMP1), truncated	1	
95	PFB0976w	874963	875163	200	1819	hypothetical protein (= PF10_0386)	9	
96	PFB0980w	876982	877830	848	636	hypothetical protein	q-gene	
97	PFB0985c	878466	879249	783	2218	hypothetical protein	pfmc-2tm	
98	PFB0990c	881467	882265	798	1272	hypothetical protein	o-gene	
99	PFB0995w	883537	884310	773	2605	hypothetical protein	n-gene	
100	PFB1000w	886915	887802	887	1858	rifin	2	
101	PFB1005w	889660	890745	1085	2111	rifin	2	
102	PFB1010w	892856	894206	1350	2020	rifin	2	
103	PFB1015w	896226	897420	1194	2031	rifin	2	
104	PFB1020w	899451	900445	994	1324	stevor, putative	3	
105	PFB1025w	901769	902026	257	474	erythrocyte membrane protein 1 (PfEMP1), truncated, degenerate	1	
106	PFB1030w	902500	902853	353	1493	hypothetical protein	1	
107	PFB1035w	904346	905775	1429	1903	rifin	2	
108	PFB1040w	907678	908861	1183	489	rifin	2	
109	PFB1045w	909350	911054	1704	2190	erythrocyte membrane protein 1 (PfEMP1), truncated	1	
110	PFB1050w	913244	914457	1213	1895	rifin	2	
111	PFB1055c	916352	923648	7296	10358	erythrocyte membrane protein 1 (PfEMP1)	1	
112	PFB1060w	934006	936779	2773	3165	hypothetical protein	9	
113	PFB1065c	939944	940511	567		hypothetical protein	9	
114	PFC0002c	8394	10745	2351	22896	hypothetical protein	9	
115	PFC0005w	33641	41158	7517	1887	Var Protein, putative	1	
116	PFC0010c	43045	44255	1210	2193	rifin	2	
117	PFC0015c	46448	47828	1380	1128	VARC pseudogene	1	1
118	PFC0025c	48956	49949	993	2117	stevor, putative	3	
119	PFC0030c	52066	53260	1194	4843	rifin	2	
120	PFC0035w	58103	59390	1287	1858	rifin	2	
121	PFC0040w	61248	62459	1211	2145	rifin	2	
122	PFC0045w	64604	65508	904	628	rifin-like protein	2	
123	PFC0050c	66136	68595	2459	4577	long chain fatty acid ligase, putative	13	
124	PFC0055w	73172	74152	980	721	hypothetical protein	9	
125	PFC0060c	74873	77071	2198	1618	FIKK3	11	
126	PFC0065c	78689	80193	1504	1654	alpha/beta hydrolase protein, putative	13	
127	PFC0070c	81847	82809	962	2119	hypothetical protein	7	
128	PFC0075c	84928	85909	981	1884	hypothetical protein	8	
129	PFC0080c	87793	90714	2921	2534	hypothetical protein	9	
130	PFC0085c	93248	94587	1339	6418	hypothetical protein, conserved	7	
131	PFC0090w	101005	101961	956	632	hypothetical protein, conserved	8	
132	PFC0095c	102593	103789	1196		hypothetical protein, conserved	9	
133	PFC1070c	999860	1000734	874	1888	VARC pseudogene	1	1
134	PFC1075w	1002622	1003470	848	613	hypothetical protein	q-gene	
135	PFC1080c	1004083	1004872	789	2140	hypothetical protein, conserved in P. falciparum	pfmc-2tm	

Rank	PlasmoDB ID	location	location	size	upstream	annotation	Group	Pseudogene
136	PFC1085c	1007012	1007810	798	1215	hypothetical protein, conserved	o-gene	
137	PFC1090w	1009025	1010134	1109	2331	hypothetical protein, conserved in P. falciparum	n-gene	
138	PFC1095w	1012465	1013612	1147	1932	rifin (3D7-rifT3-5)	2	
139	PFC1100w	1015544	1016643	1099	2025	rifin (3D7-rifT3-6)	2	
140	PFC1105w	1018668	1019678	1010	1046	stevor (3D7-stevorT3-2)	3	
141	PFC1110w	1020724	1022081	1357	2160	VARC pseudogene	1	1
142	PFC1115w	1024241	1025599	1358	1893	rifin (3D7-rifT3-7)	2	
143	PFC1120c	1027492	1034924	7432	11937	var (3D7-varT3-2)	1	
144	PFC1125w	1046861	1050978	4117		hypothetical protein	9	
145	PFD0005w	35153	44124	8971	4355	erythrocyte membrane protein 1(PfEMP1)	1	
146	PFD0015c	48479	49821	1342	2181	rifin	2	
147	PFD0020c	52002	63307	11305	2776	erythrocyte membrane protein 1(PfEMP1)	1	
148	PFD0025w	66083	67386	1303	2165	rifin	2	
149	PFD0030c	69551	70923	1372	2125	rifin	2	
150	PFD0035c	73048	74020	972	2388	stevor	3	
151	PFD0040c	76408	77560	1152	2155	rifin	2	
152	PFD0045c	79715	80787	1072	4853	rifin	2	
153	PFD0050w	85640	86898	1258	1796	rifin	2	
154	PFD0055w	88694	90022	1328	1822	rifin	2	
155	PFD0060w	91844	93055	1211	2545	rifin	2	
156	PFD0065w	95600	96631	1031	2392	STEVOR pseudogene	3	1
157	PFD0070c	99023	100256	1233	6878	rifin	2	
158	PFD0075w	107134	108560	1426	1632	hypothetical protein	9	
159	PFD0080c	110192	112053	1861	2777	hypothetical protein	5	
160	PFD0085c	114830	117745	2915	3307	ATP-dept. acyl-coa synthetase, putative	13	
161	PFD0090c	121052	122509	1457		hypothetical protein	4	
162	PFD1120c	1075873	1076283	410	4503	etramp / sep	14	
163	PFD1130w	1080786	1081973	1187	392	hypothetical protein	9	
164	PFD1135c	1082365	1082927	562	2471	hypothetical protein	9	
165	PFD1140w	1085398	1086628	1230	174	hypothetical protein	8	
166	PFD1145c	1086802	1088589	1787	1830	hypothetical protein	9	
167	PFD1150c	1090419	1095512	5093	2066	hypothetical protein	9	
168	PFD1155w	1097578	1102275	4697	1976	erythrocyte binding antigen, putative	13	
169	PFD1160w	1104251	1111551	7300	2138	SURFIN, surface-associated interspersed gene	10	
170	PFD1165w	1113689	1115922	2233	1052	FIKK4.1	11	
171	PFD1170c	1116974	1118115	1141	3944	RESA-like protein, truncated	5	
172	PFD1175w	1122059	1126043	3984	1571	FIKK4.2 /R45	11	
173	PFD1180w	1127614	1128632	1018	2774	trophozoite antigen r45-like protein,truncated	5	
174	PFD1185w	1131406	1132429	1023	858	hypothetical protein	4	
175	PFD1190c	1133287	1133655	368	540	hypothetical protein	9	
176	PFD1195c	1134195	1134520	325	3522	hypothetical protein	9	
177	PFD1200c	1138042	1138802	760	1411	hypothetical protein	7	
178	PFD1205w	1140213	1141012	799	4197	hypothetical integral membrane protein	9	
179	PFD1210w	1145209	1145718	509	1896	hypothetical protein	4	
180	PFD1215w	1147614	1148446	832	1848	hypothetical protein	4	
181	PFD1220c	1150294	1151319	1025	4807	STEVOR	3	

Rank	PlasmoDB ID	location	location	size	upstream	annotation	Group	Pseudogene
182	PFD1200w	1152896	1153810	914	6235	RIF pseudogene, RIFIN pseudogene	2	1
183	PFD1230c	1156126	1157255	1129	2790	RIFIN	2	
184	PFD1235w	1160045	1171443	11398	2159	erythrocyte membrane protein 1 (PfEMP1)	1	
185	PFD1240w	1173602	1174806	1204	1869	RIFIN	2	
186	PFD1245c	1176675	1183848	7173	8906	erythrocyte membrane protein 1 (PfEMP1)	1	
187	PFD1250w	1192754	1195747	2993		hypothetical protein, conserved in P falciparum	9	
188	PFE0005w	20929	28456	7527	777	erythrocyte membrane protein 1 (PfEMP1)	1	
189	PFE0010c	29233	29304	71	619	VAR pseudogene	1	1
190	PFE0015c	29923	30963	1040	2093	RIF pseudogene	2	1
191	PFE0020c	33056	34378	1322	2086	rifin	2	
192	PFE0025c	36464	37576	1112	1867	rifin	2	
193	PFE0030c	39443	40488	1045	641	STEVOR pseudogene	3	1
194	PFE0035c	41129	41403	274	1924	RIF pseudogene	2	1
195	PFE0040c	43327	47761	4434	2661	MESA / PfEMP2	13	
196	PFE0045c	50422	52510	2088	3331	FIKK5	11	
197	PFE0050w	55841	56872	1031	1082	hypothetical protein	8	
198	PFE0055c	57954	59726	1772	4339	heat shock protein, putative	12	
199	PFE0060w	64065	65489	1424	3441	hypothetical protein	6	
200	PFE0065w	68930	70113	1183	4396	skeleton binding protein	13	
201	PFE0070w	74509	79842	5333		interspersed repeat antigen, putative	13	
202	PFE1590w	1301219	1301764	545	1893	etramp	14	
203	PFE1595c	1303657	1304521	864	3752	hypothetical protein	9	
204	PFE1600w	1308273	1309935	1662	2531	hypothetical protein	5	
205	PFE1605w	1312466	1314149	1683	2188	protein with DNAJ domain	5	
206	PFE1610w	1316337	1316841	504	1685	hypothetical protein	8	
207	PFE1615c	1318526	1318984	458	3411	hypothetical protein	8	
208	PFE1620c	1322395	1322718	323	77	VAR fragment, pseudogene	1	1
209	PFE1625c	1322795	1323082	287	3158	VAR fragment, pseudogene	1	1
210	PFE1630w	1326240	1327424	1184	2172	rifin	2	
211	PFE1635w	1329596	1330803	1207	2662	RIF pseudogene	2	1
212	PFE1640w	1333465	1342959	9494		erythrocyte membrane protein 1(PfEMP1),truncated, pseudogene	1	1
213	PFF0005c	653	1432	779	2071	PfEMP1, truncated (pseudogene)	1	1
214	PFF0010w	3503	12835	9332	2230	erythrocyte membrane protein 1(PfEMP1)	1	
215	PFF0015c	15065	16410	1345	2176	rifin	2	
216	PFF0020c	18586	22721	4135	3836	PfEMP1-like protein	1	
217	PFF0025w	26557	27830	1273	3038	rifin	2	
218	PFF0030c	30868	31484	616	481	PfEMP1, truncated (pseudogene)	1	1
219	PFF0035c	31965	33244	1279	2111	rifin	2	
220	PFF0040c	35355	36613	1258	2108	RIFIN, frameshifted (pseudogene)	2	1
221	PFF0045c	38721	39781	1060	2316	RIF, pseudogene	2	1
222	PFF0050c	42097	43218	1121	1229	hypothetical protein, conserved in P. falciparum	n-gene	
223	PFF0055w	44447	45213	766	2247	hypothetical protein, conserved in P. falciparum	o-gene	
224	PFF0060w	47460	48255	795	616	hypothetical protein, conserved in P. falciparum	pfmc-2tm	
225	PFF0065c	48871	49719	848	1629	hypothetical protein, conserved in P. falciparum	q-gene	
226	PFF0070w	51348	52895	1547	12890	PfEMP1, internal fragment (pseudogene)	1	1
227	PFF0075c	65785	67392	1607	1438	hypothetical protein	5	

Rank	PlasmoDB ID	location	location	size	upstream	annotation	Group	Pseudogene
228	PFF0080c	68830	69690	860	1255	hypothetical protein, conserved	9	
229	PFF0085w	70945	71932	987	6255	hypothetical protein	4	
230	PFF0090w	78187	78781	594	1219107	hypothetical protein	9	
231	PFF1510w	1297888	1299376	1488	14335	hypothetical protein	5	
232	PFF1515c	1313711	1315256	1545	1620	VAR pseudogene	1	1
233	PFF1520w	1316876	1317724	848	609	hypothetical protein, conserved in P. falciparum	q-gene	
234	PFF1525c	1318333	1319119	786	2204	hypothetical protein, conserved in P. falciparum	pfmc-2tm	
235	PFF1530c	1321323	1322097	774	1200	hypothetical protein, conserved in P. falciparum	o-gene	
236	PFF1535w	1323297	1324438	1141	2544	hypothetical protein, conserved in P. falciparum	n-gene	
237	PFF1540w	1326982	1327947	965	1551	rifin (pseudogene)	2	1
238	PFF1545w	1329498	1330966	1468	2071	RIFIN	2	
239	PFF1550w	1333037	1334038	1001	2111	stevor	3	
240	PFF1555w	1336149	1337476	1327	745	rifin	2	
241	PFF1560c	1338221	1339420	1199	1890	rifin	2	
242	PFF1565c	1341310	1342598	1288	4746	rifin	2	
243	PFF1570w	1347344	1348574	1230	2153	rifin	2	
244	PFF1575w	1350727	1352090	1363	1858	rifin	2	
245	PFF1580c	1353948	1366431	12483	1482	erythrocyte membrane protein 1(PfEMP1)	1	
246	PFF1585w	1367913	1369105	1192	2397	rifin (pseudogene)	2	1
247	PFF1590w	1371502	1372862	1360	1936	RIFIN	2	
248	PFF1595c	1374798	1382628	7830		erythrocyte membrane protein 1(PfEMP1)	1	
249	MAL7P1.212	30673	38328	7655	1862	erythrocyte membrane protein 1 (PfEMP1)	1	
250	MAL7P1.213	40190	41426	1236	2263	RIFIN	2	
251	MAL7P1.214	43689	44791	1102	1196	VAR pseudogene fragment	1	1
252	MAL7P1.215	45987	47238	1251	4808	RIFIN	2	
253	MAL7P1.216	52046	53305	1259	2026	RIFIN	2	
254	MAL7P1.217	55331	56518	1187	2156	RIFIN	2	
255	MAL7P1.218	58674	59684	1010	2142	STEVOR	3	
256	MAL7P1.219	61826	62909	1083	1702	RIFIN	2	
257	MAL7P1.220	64611	65302	691	1666	hypothetical protein, conserved in P. falciparum	4	
258	MAL7P1.310	66968	68132	1164	2124	Stevor, putative, degenerate	3	
259	MAL7P1.222	70256	71400	1144	1469	RIFIN	2	
260	MAL7P1.223	72869	74407	1538	1675	STEVOR, putative	3	
261	MAL7P1.224	76082	76953	871	3878	hypothetical protein, conserved in P. falciparum	4	
262	MAL7P1.225	80831	81798	967	4592	hypothetical protein, conserved in P. falciparum	4	
263	MAL7P1.226	86390	87344	954	2248	RIFIN, pseudogene	2	1
264	MAL7P1.227	89592	90621	1029	1514	STEVOR	3	
265	MAL7P1.230	92135	93136	1001	5819	hypothetical protein, comserved in Plasmodium falciparum	9	1
266	MAL7P1.321	98955	100278	1323	5560	hypothetical protein	9	
267	MAL7P1.228	105838	108528	2690	1866	Heat Shock 70 KDa Protein, (HSP70)	12	
268	MAL7P1.229	110394	115698	5304	2023	Cytoadherence linked asexual protein (Clag)	13	
269	MAL7P1.200	117721	118781	1060	2325	RIFIN	2	
270	MAL7P1.3	121106	122238	1132	1212	hypothetical protein, conserved in P. falciparum	n-gene	
271	MAL7P1.4	123450	124218	768	2209	hypothetical protein, conserved in P. falciparum	o-gene	
272	MAL7P1.5	126427	127238	811	617	hypothetical protein, conserved in P. falciparum	pfmc-2tm	
273	PF07_0002	127855	128703	848	2593	hypothetical protein, conserved in P. falciparum	q-gene	

Rank	PlasmoDB ID	location	location	size	upstream	annotation	Group	Pseudogene
274	PF07_0003	131296	132433	1137		RIFIN	2	
275	MAL7P1.170	1376684	1377745	1061	3008	ring stage expressed protein	13	
276	MAL7P1.171	1380753	1387191	6438	7146	hypothetical protein	8	
277	MAL7P1.172	1394337	1397168	2831	1052	hypothetical protein	8	
278	MAL7P1.173	1398220	1399374	1154	6036	hypothetical protein	7	
279	MAL7P1.174	1405410	1406527	1117	2609	hypothetical protein	5	
280	MAL7P1.175	1409136	1410860	1724	2573	FIKK7.2	11	1
281	MAL7P1.176	1413433	1417946	4513	5593	erythrocyte binding antigen	13	
282	PF07_0129	1423539	1425974	2435	1467	ATP-dept. acyl-coa synthetase	13	
283	MAL7P1.177	1427441	1428138	697	6168	predicted integral membrane protein, conserved in P. falciparum	7	
284	MAL7P1.178	1434306	1436147	1841	4866	hypothetical protein (= PF10_0018, PF10_0020, PF13_0153)	9	
285	PF07_0130	1441013	1442004	991	1127	stevor	3	
286	MAL7P1.183	1443131	1444415	1284	1674	VAR pseudogene	1	1
287	PF07_0132	1446089	1447421	1332	4394	Rifin	2	
288	MAL7P1.184	1451815	1453017	1202	1813	rifin	2	
289	PF07_0134	1454830	1456060	1230	1941	rifin	2	
290	MAL7P1.185	1458001	1459356	1355	2875	rifin	2	
291	MAL7P1.350	1462231	1462440	209	1719	hypothetical protein	4	
292	PF07_0136	1464159	1465352	1193	461	rifin	2	
293	MAL7P1.186	1465813	1467609	1796	2203	Var-like	1	
294	PF07_0138	1469812	1471061	1249	1905	rifin	2	
295	MAL7P1.187	1472966	1481612	8646		erythrocyte membrane protein 1(PfEMP1)	1	
296	MAL8P1.164	9315	12632	3317	9737	hypothetical protein conserved	9	
297	PF08_0142	22369	29661	7292	1047	erythrocyte membrane protein 1 (PfEMP1)	1	
298	PF08_0141	30708	40041	9333	1915	erythrocyte membrane protein 1 (PfEMP1)	1	
299	PF08_0140	41956	51947	9991	1820	erythrocyte membrane protein 1 (PfEMP1)	1	
300	PF08_0139	53767	55102	1335	2374	RIFIN	2	
301	PF08_0138	57476	58827	1351	3435	RIFIN	2	
302	MAL8P1.163	62262	63259	997	3581	hypothetical protein	4	
303	MAL8P1.162	66840	72909	6069	8038	SURFIN, surface-associated interspersed gene	10	
304	MAL8P1.161	80947	81766	819	2195	hypothetical protein	6	
305	MAL8P1.160	83961	85028	1067	664	hypothetical protein	9	
306	PF08_0137	85692	89546	3854		hypothetical protein	9	
307	PF08_0002	1311708	1318026	6318	645	SURFIN, surface-associated interspersed gene	10	
308	PF08_0001	1318671	1319412	741	3076	hypothetical protein	8	
309	MAL8P1.2	1322488	1323896	1408	2319	hypothetical protein with DNAJ domain	5	
310	MAL8P1.1	1326215	1332258	6043	2061	SURFIN, surface-associated interspersed gene	10	
311	MAL8P1.204	1334319	1337370	3051	3161	hypothetical protein	9	
312	MAL8P1.205	1340531	1343377	2846	2099	hypothetical protein	8	
313	MAL8P1.206	1345476	1346558	1082	2570	hypothetical protein, conserved in P. falciparum	7	
314	MAL8P1.207	1349128	1360526	11398	2157	erythrocyte membrane protein 1, PfEMP1	1	
315	MAL8P1.208	1362683	1364025	1342	1880	RIFIN	2	
316	MAL8P1.209	1365905	1366306	401	661	VAR-like protein	1	
317	MAL8P1.210	1366967	1367167	200	70	hypothetical protein, conserved in P. falciparum	9	
318	MAL8P1.211	1367237	1367455	218	1254	hypothetical protein, conserved in P. falciparum	9	
319	MAL8P1.212	1368709	1369392	683	59	RESA-like pseudogene	13	1

Rank	PlasmoDB ID	location	location	size	upstream	annotation	Group	Pseudogene
320	MAL8P1.320	1369451	1369762	311	673	hypothetical protein	q-gene	
321	MAL8P1.213	1370435	1371241	806	1696	hypothetical protein, conserved in P. falciparum	pfmc-2tm	
322	MAL8P1.214	1372937	1373982	1045	1419	Stever pseudogene, putative	3	1
323	MAL8P1.215	1375401	1376349	948	1739	hypothetical protein, conserved in P. falciparum	4	
324	MAL8P1.216	1378088	1379253	1165	1942	hypothetical protein, conserved in P. falciparum	2	
325	MAL8P1.217	1381195	1382619	1424	2118	STEVOR	3	
326	MAL8P1.218	1384737	1385956	1219	2316	RIFIN	2	
327	MAL8P1.219	1388272	1389444	1172	2014	RIFIN	2	
328	MAL8P1.220	1391458	1399234	7776	8634	erythrocyte membrane protein 1 (PfEMP1)	1	
329	MAL8P1.335	1407868	1410774	2906	2006	hypothetical protein, conserved in P. falciparum	9	
330	MAL8P1.330	1412780	1413400	620		hypothetical protein	9	
331	PFI0002w	4171	4620	449	15460	hypothetical proein conserved in P. falciparum	9	
332	PFI0005w	20080	27885	7805	1912	erythrocyte membrane protein 1 (PfEMP1)	1	
333	PFI0010c	29797	31157	1360	2396	RIFIN	2	
334	PFI0015c	33553	34795	1242	4759	RIFIN	2	
335	PFI0020w	39554	40740	1186	2169	RIFIN	2	
336	PFI0025c	42909	44045	1136	1857	RIFIN	2	
337	PFI0030c	45902	47236	1334	2376	RIFIN	2	
338	PFI0035c	49612	50971	1359	1677	RIFIN	2	
339	PFI0040c	52648	54024	1376	1050	VARC-like pseudogene	1	1
340	PFI0045c	55074	56060	986	2102	stevor	3	
341	PFI0050c	58162	59240	1078	2102	RIFIN	2	
342	PFI0055c	61342	62553	1211	1663	RIFIN	2	
343	PFI0060c	64216	64428	212	2906	hypothetical protein	4	
344	PFI0065w	67334	68625	1291	1896	RIFIN	2	
345	PFI0070w	70521	71738	1217	2671	RIFIN	2	
346	PFI0075w	74409	75746	1337	2115	RIFIN	2	
347	PFI0080w	77861	78874	1013	3128	stevor	3	
348	PFI0085c	82002	82594	592	4179	hypothetical protein	n-gene	
349	PFI0090c	86773	88074	1301	1286	hypothetical protein	9	
350	PFI0095c	89360	91294	1934	2535	FIKK9.1	11	
351	PFI0100c	93829	95612	1783	2789	FIKK9.2	11	
352	PFI0105c	98401	100338	1937	2153	FIKK9.3	11	
353	PFI0110c	102491	104592	2101	2063	FIKK9.4	11	
354	PFI0115c	106655	108405	1750	928	FIKK9.5	11	
355	PFI0120c	109333	111328	1995	2080	FIKK9.6	11	
356	PFI0125c	113408	115548	2140	3712	FIKK9.7	11	
357	PFI0130c	119260	120748	1488	872	hypothetical protein	5	
358	PFI0135c	121620	125005	3385	2410	papain family cysteine protease, putative	13	
359	PFI0140w	127415	127723	308		hypothetical protein	9	
360	PFI1730w	1413829	1419743	5914	729	cytoadherence linked asexual protein (CLAG)	13	
361	PFI1735c	1420472	1422796	2324	4667	hypothetical protein (REX 1)	9	
362	PFI1740c	1427463	1428011	548	2585	hypothetical protein (REX 2)	9	
363	PFI1745c	1430596	1431135	539	2186	hypothetical protein	9	
364	PFI1750c	1433321	1434097	776	2579	hypothetical protein	8	
365	PFI1755c	1436676	1437970	1294	2802	hypothetical protein (REX 3)	8	

Rank	PlasmoDB ID	location	location	size	upstream	annotation	Group	Pseudogene
366	PFI1760w	1440772	1441649	877	2607	hypothetical protein (REX 4)	8	
367	PFI1765c	1444256	1444835	579	2082	hypothetical protein	8	
368	PFI1770w	1446917	1447935	1018	6475	hypothetical protein	8	
369	PFI1775w	1454410	1455708	1298	2672	hypothetical protein	9	
370	PFI1780w	1458380	1459850	1470	2538	hypothetical protein	8	
371	PFI1785w	1462388	1463647	1259	2455	hypothetical protein	8	
372	PFI1790w	1466102	1467043	941	1491	hypothetical protein	5	
373	PFI1795c	1468534	1469405	871	2434	hypothetical protein	6	
374	PFI1800w	1471839	1472912	1073	1507	enzyme, putative	9	
375	PFI1805w	1474419	1475692	1273	1822	rifin	2	
376	PFI1810w	1477514	1478724	1210	2211	rifin	3	
377	PFI1815c	1480935	1482199	1264	3859	rifin	2	
378	PFI1820w	1486058	1490175	4117	2186	erythrocyte membrane protein 1(PfEMP1)	1	
379	PFI1825w	1492361	1493696	1335	1871	rifin	2	
380	PFI1830c	1495567	1503324	7757		erythrocyte membrane protein 1(PfEMP1)	1	
381	PF10_0001	28491	36165	7674	1886	erythrocyte membrane protein 1(PfEMP1)	1	
382	PF10_0002	38051	39004	953	2770	rifin	2	
383	PF10_0003	41774	42964	1190	1853	rifin	2	
384	PF10_0004	44817	46134	1317	1919	rifin	2	
385	PF10_0005	48053	50279	2226	1071	rifin	2	
386	PF10_0006	51350	52600	1250	1943	rifin	2	
387	PF10_0007	54543	54770	227	354	hypothetical protein	4	
388	PF10_0008	55124	55508	384	2461	hypothetical protein	4	
389	PF10_0009	57969	58981	1012	1087	pseudogene, stevor, putative	3	1
390	PF10_0011	60068	60777	709	112	erythrocyte membrane protein 1 (PfEMP1) pseudogene	1	1
391	PF10_0012	60889	61366	477	1787	erythrocyte membrane protein 1 (PfEMP1) pseudogene	1	1
392	PF10_0013	63153	64033	880	3775	hypothetical protein	8	
393	PF10_0014	67808	68389	581	301	hypothetical protein	4	
394	PF10_0015	68690	68962	272	1586	acyl CoA binding protein, putative	13	
395	PF10_0016	70548	70820	272	2115	acyl CoA binding protein, putative	13	
396	PF10_0017	72935	73918	983	744	hypothetical protein	4	
397	PF10_0018	74662	77573	2911	3844	hypothetical protein	8	
398	PF10_0019	81417	81740	323	4799	etramp / sep	14	
399	PF10_0020	86539	89010	2471	3564	hypothetical protein	8	
400	PF10_0021	92574	93527	953	2587	hypothetical protein	8	
401	PF10_0022	96114	96563	449	2817	hypothetical protein	9	
402	PF10_0023	99380	100362	982	2757	hypothetical protein	6	
403	PF10_0024	103119	104684	1565	1970	hypothetical protein	9	
404	PF10_0025	106654	108663	2009	4267	PF70 protein	13	
405	PF10_0026	112930	116016	3086	1448589	hypothetical protein	9	
406	PF10_0378	1564605	1567485	2880	2113	hypothetical protein	8	
407	PF10_0379	1569598	1570677	1079	4821	phospholipase, putative	13	
408	PF10_0380	1575498	1578604	3106	2245	FIKK10.2 / R45	11	
409	PF10_0381	1580849	1582212	1363	49	hypothetical protein	8	
410	PF10_0382	1582261	1582737	476	5750	hypothetical protein	9	
411	PF10_0383	1588487	1589500	1013	4441	hypothetical protein, conserved (= PF11_0033)	9	

Rank	PlasmoDB ID	location	location	size	upstream	annotation	Group	Pseudogene
412	PF10_0384	1593941	1594165	224	328	hypothetical protein	1	
413	PF10_0385	1594493	1594759	266	154	PfEMP1 truncated, putative	1	
414	PF10_0386	1594913	1595113	200	70	hypothetical protein	9	
415	PF10_0387	1595183	1595407	224	1528	hypothetical protein	9	
416	PF10_0388	1596935	1597450	515	46	hypothetical protein	q-gene	
417	PF10_0389	1597496	1597762	266	605	hypothetical protein	q-gene	
418	PF10_0390	1598367	1598933	566	2359	hypothetical protein	pfmc-2tm	
419	PF10_0391	1601292	1602066	774	1210	hypothetical protein	o-gene	
420	PF10_0392	1603276	1604394	1118	2295	hypothetical protein	n-gene	
421	PF10_0393	1606689	1607866	1177	1858	rifin	2	
422	PF10_0394	1609724	1610778	1054	1941	rifin	2	
423	PF10_0395	1612719	1613742	1023	2110	stevor, putative	3	
424	PF10_0396	1615852	1617211	1359	2108	rifin	2	
425	PF10_0397	1619319	1620524	1205	2069	rifin	2	
426	PF10_0398	1622593	1623859	1266	2016	rifin	2	
427	PF10_0399	1625875	1627033	1158	2124	rifin	2	
428	PF10_0400	1629157	1630473	1316	2088	rifin	2	
429	PF10_0401	1632561	1633613	1052	2090	rifin	2	
430	PF10_0402	1635703	1636812	1109	2375	rifin	2	
431	PF10_0403	1639187	1640454	1267	1931	rifin	2	
432	PF10_0404	1642385	1643568	1183	2392	rifin	2	
433	PF10_0405	1645960	1647307	1347	1883	rifin	2	
434	PF10_0406	1649190	1656737	7547		erythrocyte membrane protein 1(PfEMP1)	1	
435	PF11_0001	5848	6090	242	731	hypothetical protein	9	
436	PF11_0002	6821	7039	218	721	hypothetical protein	9	
437	PF11_0003	7760	8149	389	3492	hypothetical protein	9	
438	PF11_0004	11641	12734	1093	4314	hypothetical protein	9	
439	PF11_0005	17048	17203	155	1344	hypothetical protein	9	
440	PF11_0006	18547	18801	254	5359	hypothetical protein	9	
441	PF11_0007	24160	31598	7438	1068	erythrocyte membrane protein 1(PfEMP1)	1	
442	PF11_0008	32666	42386	9720	2670	erythrocyte membrane protein 1(PfEMP1)	1	
443	PF11_0009	45056	46233	1177	1879	rifin	2	
444	PF11_0010	48112	49307	1195	2316	rifin	2	
445	PF11_0011	51623	52751	1128	1672	rifin	2	
446	PF11_0012	54423	54878	455	1914	hypothetical protein, truncated	4	
447	PF11_0013	56792	57840	1048	1558	stevor, putative, degenerate	3	
448	PF11_0014	59398	60328	930	600	hypothetical protein	pfmc-2tm	
449	PF11_0015	60928	61350	422	150	hypothetical protein	q-gene	
450	PF11_0016	61500	61718	218	2723	hypothetical protein	q-gene	
451	PF11_0522	64441	64842	401	1844	pseudogene, erythrocyte membrane protein 1(PfEMP1), truncate(	1	
452	PF11_0529	66686	67965	1279	2116	rifin	2	
453	PF11_0020	70081	71289	1208	1955	rifin	2	
454	PF11_0021	73244	74442	1198	2378	rifin	2	
455	PF11_0022	76820	78000	1180	2309	pseudogene, rifin, degenerate, putative	2	1
456	PF11_0023	80309	80878	569	1768	hypothetical protein	n-gene	
457	PF11_0024	82646	83420	774	2214	hypothetical protein	o-gene	

Rank	PlasmoDB ID	location	location	size	upstream	annotation	Group	Pseudogene
458	PF11_0025	85634	86423	789	623	hypothetical protein	pfmc-2tm	
459	PF11_0026	87046	87894	848	2557	hypothetical protein	q-gene	
460	PF11_0523	90451	90879	428	2821	erythrocyte membrane protein 1(PfEMP1), truncated	1	1
461	PF11_0032	93700	94028	328	307	hypothetical protein	9	
462	PF11_0033	94335	97225	2890	5748	hypothetical protein	9	
463	PF11_0034	102973	104851	1878	2793	hypothetical protein	8	
464	PF11_0035	107644	108618	974	3590	hypothetical protein	9	
465	PF11_0036	112208	114190	1982	2839	hypothetical protein, conserved	9	
466	PF11_0037	117029	119123	2094	2312	hypothetical protein	5	
467	PF11_0038	121435	122353	918	4053	hypothetical protein	6	
468	PF11_0039	126406	126681	275	2639	etramp / sep	14	
469	PF11_0040	129320	129604	284		etramp / sep	14	
470	PF11_0503	1931243	1932492	1249	6098	hypothetical protein	8	
471	PF11_0504	1938590	1939498	908	3190	hypothetical protein	9	
472	PF11_0505	1942688	1942957	269	4163	hypothetical protein	9	
473	PF11_0506	1947120	1948832	1712	281	hypothetical protein	1	
474	PF11_0507	1949113	1965636	16523	3674	antigen 332, putative	13	
475	PF11_0508	1969310	1970784	1474	2533	hypothetical protein	8	
476	PF11_0509	1973317	1976714	3397	2462	ring-infected erythrocyte surface antigen, putative	13	
477	PF11_0510	1979176	1981157	1981	1623	FIKK11	11	
478	PF11_0511	1982780	1983346	566	4923	hypothetical protein	9	
479	PF11_0512	1988269	1990928	2659	7532	ring-infected erythrocyte surface antigen 2, RESA-2	13	
480	PF11_0513	1998460	2000223	1763	1441	hypothetical protein	12	
481	PF11_0514	2001664	2001921	257	1695	hypothetical protein, truncated	4	
482	PF11_0515	2003616	2004739	1123	2046	rifin	2	
483	PF11_0516	2006785	2007812	1027	2485	stevor, putative	3	
484	PF11_0517	2010297	2011226	929	1336	rifin	2	
485	PF11_0518	2012562	2013419	857	2205	rifin, putative,truncated	2	1
486	PF11_0519	2015624	2016877	1253	1887	rifin	2	
487	PF11_0520	2018764	2019944	1180	2783	rifin	2	
488	PF11_0521	2022727	2032796	10069		erythrocyte membrane protein 1(PfEMP1)	1	
489	PFL0005w	16973	24497	7524	1824	erythrocyte membrane protein 1(PfEMP1)	1	
490	PFL0010c	26321	27687	1366	2391	rifin	2	
491	PFL0015c	30078	31261	1183	1442	rifin	2	
492	PFL0020w	32703	41940	9237	1860	erythrocyte membrane protein 1(PfEMP1)	1	
493	PFL0025c	43800	45106	1306	1682	rifin	2	
494	PFL0030c	46788	56805	10017	4751	erythrocyte membrane protein 1(PfEMP1)	1	
495	PFL0035c	61556	64336	2780	4472	octapeptide-repeat antigen, putative	13	
496	PFL0040c	68808	70867	2059	2362	FIKK12	11	
497	PFL0045c	73229	74522	1293	2324	hypothetical protein	8	
498	PFL0050c	76846	79067	2221	2382	hypothetical protein	8	
499	PFL0055c	81449	84451	3002	5483	protein with DNAJ domain (resa-like), putative	12	
500	PFL0060w	89934	90749	815	1493	hypothetical protein	8	
501	PFL0065w	92242	92562	320	1019	hypothetical protein	9	
502	PFL0070c	93581	97146	3565		hypothetical protein	8	
503	PFL2525c	2145250	2146600	1350	3938	hypothetical protein	9	

Rank	PlasmoDB ID	location	location	size	upstream	annotation	Group	Pseudogene
504	PFL2530w	2150538	2151899	1361	2703	hypothetical protein	9	
505	PFL2535w	2154602	2156108	1506	3449	RESA-like protein, putative	13	
506	PFL2540w	2159557	2161599	2042	1092	hypothetical protein	5	
507	PFL2545c	2162691	2162918	227	5312	hypothetical protein	9	
508	PFL2550w	2168230	2169743	1513	1582	hypothetical protein, conserved in P. falciparum	q-gene	
509	PFL2555w	2171325	2172332	1007	1450	hypothetical protein	4	
510	PFL2560c	2173782	2174108	326	1204	hypothetical protein	9	
511	PFL2565w	2175312	2176070	758	4332	hypothetical protein	4	
512	PFL2570w	2180402	2182852	2450	595	acyl-coa ligase antigen	13	
513	PFL2575c	2183447	2184151	704	4592	hypothetical protein	9	
514	PFL2580w	2188743	2189593	850	873	RIFIN, pseudogene	2	1
515	PFL2585c	2190466	2191753	1287	4387	RIFIN	2	
516	PFL2590w	2196140	2197105	965	1804	hypothetical protein	4	
517	PFL2595w	2198909	2199540	631	77	hypothetical protein	4	
518	PFL2600w	2199617	2199829	212	1649	hypothetical protein	4	
519	PFL2605w	2201478	2202669	1191	2038	rifin	2	
520	PFL2610w	2204707	2205697	990	2116	stevor	3	
521	PFL2615w	2207813	2209098	1285	2449	rifin	2	
522	PFL2620w	2211547	2212537	990	2117	stevor	3	
523	PFL2625w	2214654	2216002	1348	2433	rifin	2	
524	PFL2630w	2218435	2219516	1081	2323	rifin	2	
525	PFL2635w	2221839	2222831	992	2149	stevor	3	
526	PFL2640c	2224980	2226184	1204	2431	rifin	2	
527	PFL2645c	2228615	2229723	1108	2951	rifin	2	
528	PFL2650w	2232674	2232886	212	1697	hypothetical protein, truncated	4	
529	PFL2655w	2234583	2235775	1192	2418	rifin	2	
530	PFL2660w	2238193	2239400	1207	1854	rifin	2	
531	PFL2665c	2241254	2248945	7691		erythrocyte membrane protein 1(PfEMP1)	1	
532	MAL13P1.1	21467	28890	7423	1818	erythrocyte membrane protein 1 (PfEMP1)	1	
533	MAL13P1.2	30708	31984	1276	2078	RIFIN	2	
534	PF13_0003	34062	44845	10783	2841	erythrocyte membrane protein 1 (PfEMP1)	1	
535	PF13_0004	47686	48872	1186	1893	RIFIN	2	
536	PF13_0005	50765	51960	1195	2210	RIFIN	2	
537	PF13_0006	54170	55362	1192	1850	RIFIN	2	
538	MAL13P1.4	57212	58525	1313	1675	RIFIN	2	
539	MAL13P1.6	60200	61275	1075	1343	erythrocyte membrane protein 1-like	1	
540	MAL13P1.7	62618	63650	1032	2001	stevor	3	
541	MAL13P1.8	65651	66919	1268	1668	RIF pseudogene	2	1
542	MAL13P1.11a	68587	68799	212	55	hypothetical protein, conserved in P. falciparum	4	
543	MAL13P1.11	68854	69514	660	3688	hypothetical protein	4	
544	PF13_0010	73202	74516	1314	4375	Gbph2	13	
545	MAL13P1.58	78891	79681	790	3140	hypothetical protein	4	
546	PF13_0073	82821	84121	1300	1545	hypothetical protein	8	
547	MAL13P1.59	85666	86727	1061	2695	hypothetical protein	4	
548	MAL13P1.60	89422	93455	4033	6196	erythrocyte binding antigen 140	13	
549	MAL13P1.61	99651	100624	973	2321	hypothetical protein	6	

Rank	PlasmoDB ID	location	location	size	upstream	annotation	Group	Pseudogene
550	PF13_0074	102945	108482	5537	55	SURFIN, surface-associated interspersed gene	10	
551	PF13_0075	108537	109574	1037	224	SURFIN, surface-associated interspersed gene	10	
552	MAL13P1.62	109798	110371	573	2524	hypothetical protein	9	
553	PF13_0076	112895	113918	1023	7922	hypothetical protein	7	
554	PF13_0011	121840	122493	653	1437	plasmodium falciparum gamete antigen 27/25	13	
555	PF13_0012	123930	124619	689		hypothetical protein	9	
556	MAL13P1.440	2800477	2801371	894	126	hypothetical protein, pseudogene	9	1
557	MAL13P1.450	2801497	2801729	232	452	hypothetical protein, conserved in P. falciparum	9	
558	MAL13P1.455	2802181	2802348	167	292	conserved hypothetical protein	9	
559	MAL13P1.460	2802640	2803129	489	4044	conserved hypothetical protein	9	
560	MAL13P1.465	2807173	2807664	491	2282	hypothetical protein	9	
561	MAL13P1.470	2809946	2811316	1370	2731	hypothetical protein	7	
562	MAL13P1.475	2814047	2816049	2002	1684	hypothetical protein conserved in P. falciparum	5	
563	MAL13P1.480	2817733	2818697	964	4404	histidine-rich protein iii	13	
564	MAL13P1.485	2823101	2825692	2591	1003	acyl-coa ligase antigen	13	
565	MAL13P1.490	2826695	2827778	1083	1018	StevoR	3	
566	MAL13P1.495	2828796	2830233	1437	2140	RIFIN	2	
567	MAL13P1.500	2832373	2833648	1275	2428	RIFIN	2	
568	MAL13P1.505	2836076	2837076	1000	1054	STEVOR	3	
569	MAL13P1.510	2838130	2839434	1304	1699	erythrocyte membrane protein 1 (PfEMP1), pseudogene	1	1
570	MAL13P1.515	2841133	2842372	1239	1701	RIFIN	2	
571	MAL13P1.520	2844073	2845298	1225	2903	RIFIN	2	
572	MAL13P1.525	2848201	2848410	209	1708	conserved hypothetical	4	
573	MAL13P1.530	2850118	2851348	1230	2453	RIFIN	2	
574	MAL13P1.535	2853801	2855163	1362	1833	RIFIN	2	
575	MAL13P1.356	2856996	2864550	7554		erythrocyte membrane protein 1 (PfEMP1)	1	1
576	PF14_0001	1394	5344	3950	1865	erythrocyte membrane protein 1 (PfEMP1) pseudogene	1	1
577	PF14_0002	7209	8539	1330	2436	RIFIN	2	
578	PF14_0003	10975	12118	1143	2009	RIFIN	2	
579	PF14_0004	14127	15364	1237	2106	RIFIN	2	
580	PF14_0005	17470	18809	1339	2089	RIFIN	2	
581	PF14_0006	20898	22233	1335	2105	RIFIN	2	
582	PF14_0007	24338	25321	983	2001	stevor, putative	3	
583	PF14_0008	27322	28429	1107	1655	RIFIN	2	
584	PF14_0009	30084	31009	925	3685	hypothetical protein	4	
585	PF14_0010	34694	35774	1080	5516	glycophorin binding protein-related antigen	13	
586	PF14_0013	41290	43151	1861	3683	hypothetical protein	q-gene	
587	PF14_0014	46834	47745	911	1168	hypothetical protein	7	
588	PF14_0015	48913	50416	1503	2996	aminopeptidase, putative	13	
589	PF14_0016	53412	53735	323	3438	etramp / sep	14	
590	PF14_0017	57173	58294	1121	3071	lysophospholipase, putative	13	
591	PF14_0018	61365	63224	1859	3045	hypothetical protein	8	
592	PF14_0019	66269	67081	812		hypothetical protein	9	
593	PF14_0740	3167633	3169163	1530	130	hypothetical protein	8	
594	PF14_0741	3169293	3170762	1469	1280	hypothetical protein	9	
595	PF14_0742	3172042	3172714	672	2224	hypothetical protein	9	

Rank	PlasmoDB ID	location	location	size	upstream	annotation	Group	Pseudogene
596	PF14_0743	3174938	3175528	590	3166	hypothetical protein	9	
597	PF14_0744	3178694	3179680	986	3029	hypothetical protein	4	
598	PF14_0745	3182709	3183422	713	4536	hypothetical protein	4	
599	PF14_0746	3187958	3189618	1660	2737	hypothetical protein	5	
600	PF14_0747	3192355	3198548	6193	3103	SURFIN, surface-associated interspersed gene	10	
601	PF14_0748	3201651	3203087	1436	3610	hypothetical protein	4	
602	PF14_0749	3206697	3206969	272	1230	acyl CoA binding protein	13	
603	PF14_0751	3208199	3210644	2445	2007	fatty acyl coenzyme A synthetase-1, putative	13	
604	PF14_0752	3212651	3213687	1036	1782	hypothetical protein	4	
605	PF14_0753	3215469	3216366	897	2623	hypothetical protein	6	
606	PF14_0754	3218989	3219339	350	657	hypothetical protein	9	
607	PF14_0755	3219996	3220646	650	1712	hypothetical protein	6	
608	PF14_0756	3222358	3223702	1344	4040	hypothetical protein	9	
609	PF14_0757	3227742	3228659	917	3050	hypothetical protein	4	
610	PF14_0758	3231709	3235605	3896	2610	hypothetical protein	7	
611	PF14_0759	3238215	3238670	455	3309	hypothetical protein	9	
612	PF14_0760	3241979	3242699	720	10463	hypothetical protein	8	
613	PF14_0761	3253162	3255624	2462	581	fatty acyl CoA synthetase 1	13	
614	PF14_0762	3256205	3256911	706	2904	hypothetical protein	9	
615	PF14_0763	3259815	3260797	982	1816	hypothetical protein	4	
616	PF14_0764	3262613	3263271	658	55	hypothetical protein	4	
617	PF14_0765	3263326	3263538	212	1668	hypothetical protein	4	
618	PF14_0766	3265206	3266447	1241	2117	RIFIN	2	
619	PF14_0767	3268564	3269566	1002	2405	stevor, putative	3	
620	PF14_0768	3271971	3272918	947	2382	RIFIN	2	
621	PF14_0769	3275300	3276571	1271	1999	RIFIN	2	
622	PF14_0770	3278570	3279732	1162	2088	RIFIN	2	
623	PF14_0771	3281820	3282822	1002	2148	stevor, putative	3	
624	PF14_0772	3284970	3286073	1103	3950	RIFIN	2	
625	PF14_0773	3290023	3290571	548		erythrocyte membrane protein 1 (PfEMP1) pseudogene	1	1

Family	Group
Var supergene	1
Rif	2
Stevor	3
PHISTa	4
PHISTb	5
Pexel with 2TM	6
Pexel with 1TM	7
Pexel no TM	8
Hypo w/o PEXEL	9
Surfin	10
FIKK	11
Heat shock protein / DNAJ	12
Other annotated genes	13
etramp / sep	14
