# Supplementary material for: A highly conserved segmental duplication in the subtelomeres of Plasmodium falciparum chromosomes varies in copy number
Source: Malar J. 2008 Mar 7;7:46. doi: 10.1186/1475-2875-7-46 (PMC2279139; doi:10.1186/1475-2875-7-46)

Additional File 2

Topology of proteins encoded by the genes in the SDs. Red bars show transmembrane regions predicted by TMHMM; PEXEL motifs are indicated by green bars

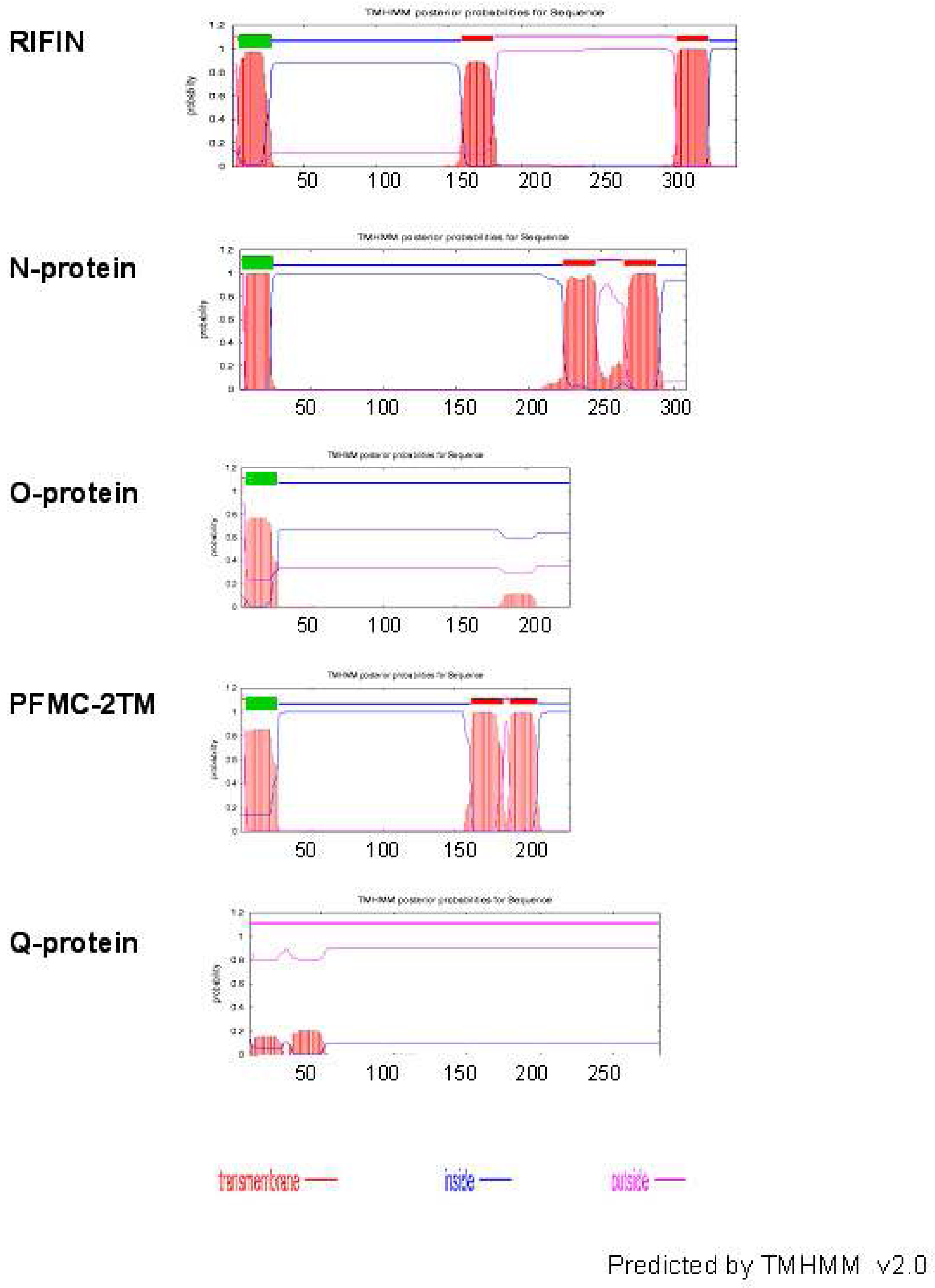

Supplement: Additional file 2 — Topology of proteins encoded by the genes in the SDs. Red bars show transmembrane regions predicted by TMHMM; PEXEL motifs are indicated by green bars. [file 1475-2875-7-46-S2.pdf]
