# Supplementary material for: A highly conserved segmental duplication in the subtelomeres of Plasmodium falciparum chromosomes varies in copy number
Source: Malar J. 2008 Mar 7;7:46. doi: 10.1186/1475-2875-7-46 (PMC2279139; doi:10.1186/1475-2875-7-46)
Supplement: Additional file 3 — SNPs in n-, o-, pfmc-2tm and q-gene found in different P.f. strains. [file 1475-2875-7-46-S3.pdf]

Additional File 3. SNPs in *n-, o-, pfmc-2tm and q-gene* found in different *P.f.* strains

Gene	Reference Gene / remarks	start	end	bp surveyed	SNP Position	NS	S	3D7	FCR3	TM180	7G8	UAS39	UAS31	7G8*	Dd2*	D10*	HB3*
n-gene	PFA0055c	69594	70343	749	69760	+		T/C	C	C	T/C	T/C	T/C	C	C	C	C
					69761	+		A/G	G	G	G	A/G	G				
					69779	+		T/G	G	T/G	G	G	G	G	G	G	G
					69947	+		A	A/G	A/G	A/G	A/G	A/G	A/G	A/G	A	A
o-gene	no SNP detected			560													
pfmc-2tm	variant gene family			544													
q-gene	PF07_0002	127855	128703	746	127990	+		G/A	G	G	G/A	G/A	G				
					128383	+		A/G	A/G	A	A	A	A/G				
					128396	+		A/G	A/G	A	A	A	A/G				
					127872		+	T	-	-	-	-	-		C		T
					127880	+		A/G	-	-	-	-	-		A		A
					127897		+	A/G	-	-	-	-	-		A		A

Sequences were manually curated by visualization of the chromatograms. Criteria for true SNPs are: (1) SNP present in at least two parasites. (2) At least two sequences showing the particular SNP in one of the parasites.
NS, non-synonymous; S, synonymous; * information acquired from [39]; - sequenced area not covered
