# Supplementary material for: A highly conserved segmental duplication in the subtelomeres of Plasmodium falciparum chromosomes varies in copy number
Source: Malar J. 2008 Mar 7;7:46. doi: 10.1186/1475-2875-7-46 (PMC2279139; doi:10.1186/1475-2875-7-46)
Supplement: Additional file 4 — Copy number estimation of SD genes using BLASTN. [file 1475-2875-7-46-S4.pdf]

**Additional File 4. Copy number estimation of SD genes using BLASTN**

| | | BLAST against the reads | | | | | BLAST against the assemblies | |
|---|---|---|---|---|---|---|---|---|
| SD Gene | Strain | bps aligned | coverage | average coverage | estimate copies # (95 % identity cutoff) | estimate copies # (90 % similarity cutoff) | on-line BLAST | Probability |
| *n-gene* | Hb3 | 100492 | 14.0 | 8.07 | 1 | 2 | 2 | < 1.2e-75 |
| | Dd2 | 31 | 0.0 | 7.13 | 1 / 0 | 1 / 0 | 1 | |
| | Ghanian Isolates | 195400 | 23.6 | 8 | 3 | 3 | N.A. | |
| | It | 85465 | 10.3 | 3.84 | 3 | 3 | 3 | |
| *o-gene* | Hb3 | 25165 | 4.0 | 8.07 | 1 | 1 | 4 | < 3.2e-98 |
| | Dd2 | 47630 | 7.6 | 7.13 | 1 | 1 | 1 | |
| | Ghanian Isolates | 126213 | 20.1 | 8 | 3 | 3 | N.A. | |
| | It | 28433 | 4.5 | 3.84 | 1 | 1 | 2 | |
| *pfmc-2tm** | Hb3 | 40122 | 6.8 | 8.07 | 6 | 7 | 9 | < 4.5e-15 |
| | Dd2 | 7354 | 1.2 | 7.13 | 2 | 3 | 3 | |
| | Ghanian Isolates | 27834 | 4.7 | 8 | 3 | 6 | N.A. | |
| | It | 2535 | 0.43 | 3.84 | 1 | 2 | 5 | |
| *q-gene* | Hb3 | 195639 | 36.7 | 8.07 | 5 | 5 | 7 | < 4.2e-40 |
| | Dd2 | 93161 | 17.5 | 7.13 | 2 | 2 | 3 | |
| | Ghanian Isolates | 185752 | 34.8 | 8 | 4 | 5 | N.A. | |
| | It | 101026 | 18.9 | 3.84 | 5 | 6 | 5 | |

# The identity cutoff was set to 95% or 90%. The reads had to have a complete overlap or an overlap that ended in the beginning or end of either the read or the gene, with the minimum length overlapping of 36 bp. No partial overlapping was allowed. N.A., information not available, due to the uncomplete sequence assembly of the IT strain, number of copy cannot be justified by online BLASTN. ***** due to the presence of hypervariable region, conserved region of the *pfmc-2tm* gene (250 bp before the first transmembrane region) was used as the input sequence for the analysis and on-line BLASTN.
